# Heat Safety in the Workplace: Modified Delphi Consensus to Establish Strategies and Resources to Protect the US Workers

**DOI:** 10.1029/2021GH000443

**Published:** 2021-08-01

**Authors:** Margaret C. Morrissey, Douglas J. Casa, Gabrielle J. Brewer, William M. Adams, Yuri Hosokawa, Courteney L. Benjamin, Andrew J. Grundstein, David Hostler, Brendon P. McDermott, Meredith L. McQuerry, Rebecca L. Stearns, Erica M. Filep, David W. DeGroot, Juley Fulcher, Andreas D. Flouris, Robert A. Huggins, Brenda L. Jacklitsch, John F. Jardine, Rebecca M. Lopez, Ronda B. McCarthy, Yannis Pitisladis, Riana R. Pryor, Zachary J. Schlader, Caroline J. Smith, Denise L. Smith, June T. Spector, Jennifer K. Vanos, W. Jon Williams, Nicole T. Vargas, Susan W. Yeargin

**Affiliations:** ^1^ Department of Kinesiology Korey Stringer Institute University of Connecticut Mansfield CT USA; ^2^ Department of Kinesiology University of North Carolina at Greensboro Greensboro NC USA; ^3^ Faculty of Sports Sciences Waseda University Saitama Japan; ^4^ Department of Kinesiology Samford University Birmingham AL USA; ^5^ Department of Geography The University of Georgia Athens GA USA; ^6^ Department of Exercise and Nutrition Sciences Center for Research and Education in Special Environments Buffalo NY USA; ^7^ Department of Health, Human Performance and Recreation University of Arkansas Fayetteville AR USA; ^8^ Retail Entrepreneurship Florida State University Tallahassee FL USA; ^9^ Fort Benning Heat Center Martin Army Community Hospital Fort Benning GA USA; ^10^ Public Citizen Washington DC USA; ^11^ Department of Exercise Science FAME Laboratory University of Thessaly Trikala Greece; ^12^ National Institute for Occupational Safety and Health Cincinnati OH USA; ^13^ School of Physical Therapy & Rehabilitation Sciences Morsani College of Medicine University of South Florida Tampa FL USA; ^14^ Medical Surveillance Services Concentra Waco TX USA; ^15^ Collaborating Centre of Sports Medicine University of Brighton Brighton UK; ^16^ Department of Kinesiology School of Public Health Indiana University Bloomington IA USA; ^17^ Department of Health and Exercise Science Appalachian State University Boone NC USA; ^18^ Department of Health and Human Physiological Sciences First Responder Health and Safety Laboratory Skidmore College Saratoga Springs NY USA; ^19^ Department of Environmental and Occupational Health Sciences School of Public Health University of Washington Seattle WA USA; ^20^ School of Sustainability Arizona State University Tempe AZ USA; ^21^ Centers for Disease Control and Prevention (CDC) National Personal Protective Technology Laboratory (NPPTL) National Institute for Occupational Safety and Health (NIOSH) Pittsburgh PA USA; ^22^ Faculty of Health Sciences University of Sydney Sydney NSW Australia; ^23^ Department of Exercise Science Arnold School of Public Health University of South Carolina Columbia SC USA

**Keywords:** heat‐related illness, occupational, heat stress, safety, heat risk management

## Abstract

The purpose of this consensus document was to develop feasible, evidence‐based occupational heat safety recommendations to protect the US workers that experience heat stress. Heat safety recommendations were created to protect worker health and to avoid productivity losses associated with occupational heat stress. Recommendations were tailored to be utilized by safety managers, industrial hygienists, and the employers who bear responsibility for implementing heat safety plans. An interdisciplinary roundtable comprised of 51 experts was assembled to create a narrative review summarizing current data and gaps in knowledge within eight heat safety topics: (a) heat hygiene, (b) hydration, (c) heat acclimatization, (d) environmental monitoring, (e) physiological monitoring, (f) body cooling, (g) textiles and personal protective gear, and (h) emergency action plan implementation. The consensus‐based recommendations for each topic were created using the Delphi method and evaluated based on scientific evidence, feasibility, and clarity. The current document presents 40 occupational heat safety recommendations across all eight topics. Establishing these recommendations will help organizations and employers create effective heat safety plans for their workplaces, address factors that limit the implementation of heat safety best‐practices and protect worker health and productivity.

## Key Points

- This document presents feasible, evidenced‐based occupational heat safety recommendations to protect workers from the dangers of heat
- A roundtable of 51 experts created 40 heat safety recommendations within eight heat safety topics: heat hygiene, hydration, heat acclimatization, environmental monitoring, physiological monitoring, body cooling, textiles and personal protective gear, and emergency action plan implementation
- Implementing feasible and effective heat safety plans in the workplace will protect worker health and mitigate productivity losses

## 1 Introduction

Approximately 13.3 million US workers performed work in extreme heat every day in July 2017 (Tanglis, 2018). The US workers are at risk of heat stress and those in certain occupations (e.g., agriculture, construction, forestry, mining, firefighting, and manufacturing) are at even greater risk (NIOSH, 2016). Heat stress is defined as exposure to heat in the form of internal heat generation (physical exertion), environmental conditions (e.g., ambient temperature, relative humidity), and/or clothing worn that result in an increase in heat storage in the body. Many workers have been at high risk of heat‐related illness and death at worksites for decades, and the conditions are being exacerbated by rising temperatures related to climate change (Moda et al., 2019). Exertional heat stroke (EHS), the most life‐threatening heat‐related illness, is considered 100% survivable when appropriate procedures are in place for the management (e.g., recognition and accurate assessment of internal body temperature) and care (e.g., aggressive, whole‐body cooling using cold‐water immersion within 30 min of collapse) of the condition (Casa et al., 2015). Although we possess the knowledge to properly manage and care for those succumbing to heat‐related illness, fatalities continue to be reported each year due to occupational heat stress (CFOI, 2018).

Heat‐related illnesses and injuries occurring in occupational settings significantly impact the worker and organization (Moda et al., 2019; NIOSH, 2016; Tanglis, 2018). A meta‐analysis collated data from 33 studies involving 13,088 workers and an additional 11 studies that included 8,076 workers, and reported that 35% of workers experienced occupational heat strain during or following the work shift, while 30% of workers reported productivity losses, respectively (Flouris et al., 2018). The increasing threat of occupational heat stress requires workplace heat safety policies and procedures that reduce the negative health effects of occupational heat stress and preserve productivity.

Many different heat safety recommendations are offered within the occupational setting to mitigate the negative consequences of heat stress (ACGIH, 2017; NIOSH, 2016; “*OSHA's campaign to prevent heat illness in outdoor workers | heat fatalities [text version] | Occupational Safety and Health Administration*,” OSHA, 2011). California, Washington, and Minnesota are the only three states in the United States that enforce Occupational Safety and Health Administration (OSHA)‐approved heat safety standards (NIOSH, 2016). At the federal level, OSHA requires that employers, “shall furnish to each of his employees employment and a place of employment which are free from recognized hazards that are causing or are likely to cause death or serious physical harm to his employees” (General Duty Clause, OSH Act, Section 5(a)(1)) (“*OSHA's campaign to prevent heat illness in outdoor workers | heat fatalities [text version] | Occupational Safety and Health Administration*,” OSHA, 2011). In 2011, OSHA also introduced a heat safety awareness campaign in partnership with the National Institute for Occupational Safety and Health (NIOSH). In 2016, the partnership updated a heat safety mobile application that provides safety recommendations based on heat index (“*OSHA's campaign to prevent heat illness in outdoor workers | heat fatalities [text version] | Occupational Safety and Health Administration*,” OSHA, 2011). Both NIOSH and American College of Governmental Industrial Hygienists (ACGIH^®^) have published comprehensive heat safety guidelines (latest updates in 2016 and 2017, respectively) to protect workers and organizations from occupational heat exposure (ACGIH, 2017; NIOSH, 2016). However, although some states have state‐specific heat standards and current federal recommendations are comprehensive, studies suggest there is limited adoption of these practices. An investigation of 84 OSHA heat enforcement cases (i.e., heat illness and fatality reports) reported that 80% of employers did not rely on national standard approaches for heat illness prevention (Tustin, Cannon, et al., 2018; Tustin, Lamson, et al., 2018). Moreover, heat enforcement cases lacked at least one or more core components of a heat safety plan (e.g., heat acclimatization [HA]) (Tustin, Cannon, et al., 2018; Tustin, Lamson, et al., 2018). Similarly, a study reported that among 25 outdoor occupational heat‐related illnesses, 14 fatalities and 11 nonfatal illnesses occurred when occupational heat exposure limits (OELs) were exceeded (Tustin, Cannon, et al., 2018; Tustin, Lamson, et al., 2018). Lack of heat safety program adoption may be due to the multiple barriers that can impede heat safety program implementation or strategies (Table 1). Many of the current recommendations offer extremely effective preventive measures that can prevent neurologic, liver, kidney, and endocrine disease and most significantly death of workers exposed to occupational stress but may lead to a net loss in productivity (Morris, Jay, et al., 2020; Morris, Levi, et al., 2020). Despite the obvious health and life saving benefits of heat stress prevention programs, this may reduce the likelihood of the employer implementing current federal recommendations due to concerns over financial losses.

**Table 1:** Examples of Barriers to Implementing Effective Heat Safety Strategies in the Workplace

Worker culture and habits
Emphasis on productivity
Legal implications^a^
Fixed work hours and schedule
Cost and feasibility of heat safety best‐practices
Lack of heat safety training

^a^ Legal implications may include screening procedures that identify high risk individuals and physiological data collection (e.g., Americans with Disabilities Act, HIPAA).

To protect the health and safety of occupational workers exposed to heat stress, heat safety stakeholders must establish strategies, resources, and feasible occupational heat safety recommendations that employers will adopt. Employers are more likely to implement effective, life‐saving heat safety plans if they are characterized as “feasible” or “cost effective” (Morris, Jay, et al., 2020; Morris, Levi, et al., 2020). Morris, Jay, et al. (2020) and Morris, Levi, et al. (2020) reported that 57% of surveyed employers identified barriers to adoption of heat safety interventions. Of the 57%, 30% reported cost and 15% reported feasibility as the perceived barrier. If the proposed heat safety recommendations can realistically be implemented with limited disruption of workers' standard working procedures (i.e., feasible), employers are more likely to adopt the safety practices. Unfortunately, some employers are focused on economic growth and productivity in lieu of safety practices that are associated with positive safety and health outcomes, despite the plethora of literature that links heat stress to productivity losses (Kjellstrom et al., 2009, 2014; Lee et al., 2018; NIOSH, 2016; Parsons, 2009). To enhance employer adoption of occupational heat safety, safety programs should provide feasible (i.e., productivity enhancing) recommendations that protect the health and safety of workers susceptible to heat stress.

Given the above, the objective of the current document was to develop concise, evidence‐based, and feasible recommendations to enhance heat safety in the workplace that protects both worker health and productivity. These recommendations serve as a common starting point for all working occupations and are intended to be tailored to specific occupations and industries. Recommendations and corresponding resources within this document are tailored to safety managers, industrial hygienists, and the employers that bear responsibility for implementing heat safety programs. These recommendations are based on scientific evidence, feasibility, and clarity to further enhance heat safety best practice adoption in occupations with inherent or unavoidable heat stress. The tables, figures, and appendices are included as strategies to help safety managers and employers tailor the recommendations to their specific work setting.

## 2 Methods

To achieve our objectives, an interdisciplinary roundtable comprised of 51 individuals with expertize in various areas associated with heat‐related illness and heat safety, was assembled to develop evidence‐based heat safety recommendations. A virtual meeting was held on December 10, 2020 to provide insight on eight topics related to heat safety: heat hygiene, hydration, HA, environmental monitoring, physiological monitoring, body cooling, textiles and personal protective gear, and emergency action plan (EAP) implementation. The eight topics were chosen based on current consensus and best practices regarding heat‐related illness (Casa et al., 2015; NIOSH, 2016).

The roundtable co‐chairs (Margaret C. Morrissey and Douglas J. Casa) selected nine individuals to serve as section chairs (the heat hygiene section had two chairs) for each topic. Section chairs were responsible for coordinating with their section members to conduct a thorough review of the literature on the topic and facilitate the creation of the recommendations. Each roundtable participant was assigned to one of the eight topics based on their areas of expertize. Each group contained 6–8 participants. The roundtable attendees were identified in August and September 2020. The roundtable meeting attendees were comprised of:

1. Twenty‐nine scientists with expertize in fields of occupational health (2), thermal physiology (25), human biometeorology (2)
2. Five representatives from governing bodies: NIOSH (2), US Army (2), US Air Force (1)
3. One worker health and safety advocate (Public Citizen)
4. Twelve safety managers responsible for safety initiatives
5. Three clinicians specializing in occupational medicine and/or heat‐related illness

### 2.1 Formulation of Recommendations

The Delphi method was utilized to comprehensively and systematically form a consensus on optimal recommendations to mitigate occupational heat strain in workers with the intention to preserve productivity (Ziglio & Alder, 1996). We chose to follow the Delphi method as it allows for the integration of opinion among multiple experts and is particularly useful in areas of limited research, such as heat‐related illness prevention strategies in occupational settings (Ziglio & Alder, 1996). The Delphi method included both an exploration and evaluation phase (Ziglio & Alder, 1996).

#### 2.1.1 Exploration Phase

A narrative review of the current literature on each of the eight topics (i.e., heat hygiene, HA, hydration, environmental monitoring, physiological monitoring, body cooling, textiles and personal protective gear, EAP) was performed by the respective working group of each section. The purpose of the review was to provide a clear background of the topic to facilitate the creation of the recommendations and generate resources/strategies for implementation of these recommendations. The narrative review was also accompanied by a subsection addressing the gaps in knowledge to influence future investigations related to each topic.

#### 2.1.2 Evaluation Phase

During the roundtable meeting, working groups for each topic met to create action‐oriented recommendations. The action‐oriented recommendations were modified as necessary within each subtopic working group prior to being collated and prepared for scoring.

Once all recommendations were prepared for scoring, the roundtable co‐chairs created an online survey. All roundtable attendees received an email with a link to the anonymous online survey (^XM^Qualtrics Online Survey Software, www.qualtrics.com) to score all recommendations and provide feedback. Roundtable attendees were instructed to score each recommendation based on their background and expertize (Ziglio & Alder, 1996). Each recommendation was scored based on three categories: *scientific evidence*, *feasibility*, and *clarity*. *Scientific evidence* was operationally defined as whether the recommendation is based on current data, theory, or other scientific evidence. *Feasibility* was operationalized as whether the recommendation was realistic to implement in occupations where heat stress is a concern. Realistic implementation included consideration of costs associated with implementation and the degree to which workers' standard working procedures would be interrupted (Morris, Jay, et al., 2020; Morris, Levi, et al., 2020). *Clarity* was operationally defined as whether the recommendation was easy to understand and clear. Each category was scored on a 9‐point scale (0–9) that has been reported in previous literature (Kroshus et al., 2019). In the 9‐point scale, “1” indicated the worst score and “9” indicated the best score. Roundtable members were also required to provide open comments for recommendations where they scored the recommendation as a 4, 5, or 6 for each category. For each recommendation, mean scores were calculated for each category (i.e., scientific evidence, feasibility, clarity). Recommendations that received an average score 7 or higher for each category were transferred to the final version of the manuscript. Recommendations receiving an average score for any one component (i.e., scientific evidence, feasibility, clarity) between 4 and 7 were revised based on feedback provided by task force members. Recommendations receiving an average score of <4 for any of the three components were discarded. Forty‐four roundtable participants filled out the Delphi method scoring survey.

The roundtable co‐chairs examined recommendations that received average scores between 4 and 7 for each category. Written comments were reviewed by the roundtable section leaders and when appropriate, recommendations were modified accordingly. After modifications were made to the recommendation, the Delphi scoring and review processes were repeated by the roundtable attendees for all recommendations scoring between 4 and 7. If any recommendations received a score between 4 and 7 in any category after the second round of scoring, the roundtable section leaders deliberated to reach a final version of each recommendation. Final deliberations were achieved through discussion among section leaders.

All recommendations across all eight topics are focused on how employers and supervisors can implement specific practices, techniques, or considerations to mitigate the negative effects of heat stress. These recommendations draw on previous recommendations presented by ACGIH, NIOSH, and OSHA and uniquely provide action‐oriented and concise steps to achieving optimal heat safety, health, and productivity. Moreover, the Delphi method was utilized to integrate interdisciplinary perspectives from experts across many different disciplines related to physiology, occupational health, and heat‐related illness (Ziglio & Alder, 1996). These recommendations were not only created from a roundtable (comprised of 51 members) but were also scored based on feasibility and scientific evidence. Recommendations that are both evidenced‐based and feasible are more likely to be adopted as they limit interruption in standard working procedures and limit cost. From this perspective, heat safety plans that are necessary to keep workers healthy and safe from the dangers of heat can also serve the “employer agenda” for productivity (i.e., will not affect productivity, will enhance productivity).

### 2.2 Strength of Recommendation Taxonomy System

The level of evidence for each recommendation was evaluated by two reviewers (Margaret C. Morrissey and Gabrielle J. Brewer) using a strength of recommendation taxonomy (SORT) (Ebell et al., 2004). The SORT taxonomy system was used in conjunction with the Delphi Method scoring of each recommendation to provide a standardized appraisal of the level evidence used to create each recommendation (Ebell et al., 2004). SORT is an appraisal system with three strength of recommendation categories (A, B, C) based on patient‐oriented outcomes. Patient‐oriented outcomes in the context of this investigation were defined as outcomes that matter to workers and help them live longer and healthier lives. This includes reduced mortality, reduce morbidity, improved quality of life, and symptom improvement. Recommendations were categorized as “Level A” if they were supported by “good quality, patient‐oriented” evidence such as evidence from high‐quality systematic reviews, meta‐analyses, and randomized controlled trials. “Level B” were characterized as “limited quality, patient‐oriented” evidence, which includes systematic reviews and meta‐analyses of lower‐quality studies with inconsistent findings, cohort studies, case‐control studies, or lower quality clinical trials. “Level B” are recommendations are supported by evidence from opinions, usual practice, and case series. Each reference in this document was also appraised using the SORT level of evidence (LOE, 1, 2, 3) taxonomy system, and are provided in Table S3 in supporting information. Definitions of LOE can be found elsewhere (Ebell et al., 2004).

## 3 Results: Recommendations

As establishing evidence‐based and feasible heat safety recommendations are essential, the recommendations presented in this document are intended to serve as a foundation to building a more resilient workforce against occupational heat stress. Following the round table meeting, 59 recommendations were originally developed. After two rounds of scoring, the Delphi method resulted in 40 recommendations across all eight topics: heat hygiene (*n* = 6), hydration (*n* = 7), HA (*n* = 4), environmental monitoring (*n* = 5), physiological monitoring (*n* = 1), body cooling (*n* = 9), textiles and personal protective equipment (PPE) (*n* = 7), and EAPs (*n* = 5) are presented in Table 2. The appraisal of each recommendation and citation used to create recommendations is presented in Tables 2 and S3, respectively.

**Table 2:** Occupational Heat Safety Recommendations Created Through Modified Delphi Method

**Recommendations**	SORT (A, B, C)^a^ [14]
**Heat hygiene**	
#1: If physical examinations are required or recommended by the workplace, the healthcare provider should utilize examination results to educate employees about the potential influence of conditions that impair their ability to tolerate heat (Table 2).	C
#2: Employers should facilitate and provide access to wellness programs to minimize heat illness risk factors.	A
#3: Occupational heat safety education and/or training for workers and supervisors should include recognition and risks of heat‐related illnesses, prevention, first aid, and emergency response procedures in a language and format that is easily understood. At minimum, heat safety training should occur annually.	B
#4: Workers and supervisors should conduct their own health status checks before starting their work shift. The health status checklist should be survey‐based and/or electronic and written in accessible language and format.	C
#5: In the absence of designated personnel to monitor workers during a shift, workers should implement a “buddy approach” where each worker is assigned a “buddy.” The “buddy” should check in with their respective partner throughout the day and monitor for potential signs/symptoms of heat‐related illness.	C
#6: Supervisors should develop timely communication strategies to inform workers of acceptable work‐to‐rest ratios and other heat mitigation strategies ahead of scheduled working shifts (e.g., strategies based on inclement weather, environmental conditions). Communication should be appropriately translated into other languages when applicable.	C
**Hydration**	
#1: Employers should prioritize fluid delivery and accessibility for their workers to prevent dehydration (i.e., access and availability to cool water, potable water in the workplace).	A
#2: Strategies for fluid replacement should be developed by the supervisor/employer. Strategies for fluid replacement should account for the individual needs of the worker, intensity and duration of work, environmental conditions, and timing of rest breaks (i.e., duration, frequency).	A
#3: Employers should incorporate hydration education into employee onboarding (i.e., job training) and these strategies and concepts should be reinforced (e.g., messaging, signage, or other informational resources) during times of high heat stress.	B
#4: Employers should develop a site‐specific dehydration risk mitigation plan that includes components related to: (a) availability and accessibility to clean, portable, fluid sources and (b) drinking fluids during rest breaks.	A
#5: Employers should identify drinking strategies for their workers to optimize hydration, minimize weight loss, promote a light‐colored urine and moderate urine frequency (i.e., >5 voids per 24‐h), prevent overdrinking, and reduce thirst sensation. Employers should also provide supervisors and employees with easy access to clean restrooms.	A
#6: Employee hydration education should include modules that focus on daily fluid needs, types of fluids that optimize hydration, health behaviors that impact hydration, and self‐assessment of hydration status including monitoring of urine color, urine frequency, thirst, and weight changes.	B
#7: Electrolyte drinks should be consumed when work conditions require heavy physical exertion in hot and/or humid conditions for more than 2 h. Otherwise, cool water is an appropriate hydration beverage.	B
**Heat acclimatization (HA)**	
#1: Employers/supervisors should create and implement a gradual, progressive HA program (5–7 days) to minimize the effects of heat stress	B
#2: Employer‐initiated HA programs that are tailored to the demands of the job, environmental conditions, clothing, and PPE should be applied to all workers new to the job (day 1–day 7) and workers returning from an extended absence (e.g., injury, medical leave).	B
#3: Workers should be acclimatized to the heat by gradually increasing their exposure to heat over a 5–7‐day period. When possible or feasible, employers should also reduce new or returning workers' exposure time and/or physical demands (i.e., lower the intensity of work compared to normal work conditions) and modify work to rest ratios for the first 5–7 days.	B
#4: Employers should provide annual training and education to workers regarding the benefits of HA, the workplace HA program, and the maintenance of HA.	B
**Environmental monitoring**	
#1: Environmental measurements should be taken on‐site—as close to the individual work site as possible—to best represent environmental heat stress.	A
#2: Comprehensive heat stress assessment and associated interventions should include information on ambient environmental conditions, work demands, clothing, PPE, and worker HA status.	A
#3: Environmental measurements for heat stress assessment should account for the influences of air temperature, humidity, wind speed, and radiant heat. Indices that incorporate or integrate the individual measurements can be used for heat stress assessment (e.g., wet bulb globe temperature).	A
#4: When using portable environmental sensors, employers should follow manufacturer specifications for set up, equilibration (i.e., time for the sensor to adjust to ambient conditions), and calibration.	A
#5: Employers should incorporate environment‐based work modifications (e.g., change in number of rest breaks) into workplace policies and procedures.	A
**Physiological monitoring**	
#1: In occupational settings where there is a risk of heat‐related illness, employers should consider employing valid and reliable physiological monitoring systems (e.g., heart rate or body temperature monitoring devices) that can be used to quantify worker heat strain in accordance with other heat stress assessment parameters, such as clothing requirements and environmental conditions.	C
**Body cooling**	
#1: Job sites should have a designated rest, cooling, and hydration center that is accessible to workers as needed (Figure 3, Table 6).	B
#2: At cooling centers, body cooling strategies should be implemented, available, and/or accessible (Figure 3).	B
#3: When personal protective gear cannot be removed while on the worksite, cooling products worn under gear (e.g., cooling vests) should be considered.	B
#4: When ambient temperatures are below 40°C (104°F), electric fans or air conditioning should be used for evaporative cooling.	B
#5: If power is not available at the worksite, cooling strategies should include portable cooling modalities (e.g., ice in coolers, water, ice towels).	B
#6: If PPE, such as headgear, helmets, or gloves, can be partially removed, worksites should provide cold towels and/or ice‐water for extremity cooling (i.e., hand and forearm immersion).	B
#7: Cooling during rest breaks should be performed (e.g., immersion, shade, hydration, removal of PPE). Cooling should be done for as long as possible to achieve optimal cooling benefits.	B
#8: Workers should utilize body cooling strategies with available cooling modalities before, during, and after the work shift to achieve optimal benefits in hot and/or humid conditions.	B
#9: Workers should be educated during on‐boarding training on the effects of body cooling.	C
**Textiles and personal protective gear**	
#1: Workers should wear personal protective clothing or equipment that is thin, is lightweight, promotes heat dissipation, and safely protects against worksite hazards (i.e., biological, electrical, physical, and chemical hazards).	B
#2: Employers should select garments with ventilated openings to deploy for heat stress relief in working conditions where biological, electrical, and chemical threats are not present.	B
#3: In hot and humid climates, employees should only wear clothing and PPE that are absolutely essential for avoiding harm while completing the specific task at hand.	C
#4: Employers should select work‐specific PPE with the appropriate fit relative to proportional body differences (i.e., designed for men vs. women) and with the least amount of bulk where appropriate.	B
#5: When selecting clothing and PPE, employers should select items that are effective, reliable, and certified (if required) to withstand hot and humid working conditions.	B
#6: During rest periods, clothing layers should be removed long enough (i.e., the entire rest period) to allow for optimal body cooling and adequate recovery prior to beginning the next work session.	B
#7: In work settings requiring physical fitness or skill testing during the hiring process (i.e., firefighting), appropriate clothing and PPE should be worn.	B
**Emergency procedures and EAPs**	
#1: Each work site needs to have an EAP that addresses medical emergencies associated with heat stress (e.g., EHS). Multiple EAPs within a company may be necessary to address various needs of different work sites.	A
#2: Employers should identify the worksite managers and medical personnel to create, manage, coordinate, and execute EAPs. The EAP should be communicated to local Emergency Medical Services and updated as applicable.	A
#3: The EAP should be disseminated, rehearsed, and reviewed annually with all staff and employees.	A
#4: Review of the work sites' EAPs should be included in new employee and supervisor onboarding training.	C
#5: After a worker experiences a heat‐related illness (e.g., EHS), a return‐to‐work protocol should be established under the direction of a physician, who is ideally familiar with exertional heat illness recovery.	B

Abbreviations: EAP, emergency action plan; EHS, exertional heat stroke; HA, heat acclimatization; PPE, personal protective equipment; SORT, strength of recommendation taxonomy.

^a^ SORT is a standardized system used to appraise recommendations based on patient‐oriented outcomes (Ebell et al., 2004). Level A: good quality patient‐oriented evidence; Level B: limited‐quality patient‐oriented evidenced; Level C: other evidence.

## 4 Narrative Review

### 4.1 Heat Hygiene

#### 4.1.1 Background and Significance

The World Health Organization defines *hygiene* as conditions and practices that help maintain health and prevent the spread of diseases (WHO, 2019, https://www.afro.who.int/health-topics/hygiene). The International Occupational Hygiene Association further defines *occupational hygien*e as anticipating, recognizing, evaluating, and controlling health hazards in the working environment with the objective of protecting worker health and well‐being and safeguarding the community at large (IOHA, n.d., https://www.ioha.net/about/occupational-hygiene). We define *heat hygiene* as managing health hazards associated with worker exposure to a hot environment and/or thermal strain. In this section, we will focus on evidence‐based heat hygiene practices during the onboarding of employees and prior to the start of a working shift, given that many of the other recommendations, including those implemented during the work shift (e.g., HA, hydration, environmental monitoring, work‐to‐rest cycles, physiological monitoring, body cooling, textiles PPE, and EAP), are then provided. Examples of heat hygiene practices include identifying workers with risk factors for heat‐related illnesses, medical surveillance (e.g., physical examination), and promoting healthy lifestyle behaviors. As certain risk factors or medical conditions increase susceptibility to heat‐related illnesses, it is important for employers to recognize these factors as they may compromise workers' health, well‐being, and work capacity in the heat.

#### 4.1.2 Current Research

A retrospective case series on heat‐related illnesses among the US workers revealed that the presence of one or more of the following conditions was often associated with heat‐related illness fatalities: (a) obesity, (b) hypertension, (c) diabetes, and (d) cardiac disease (Tustin, Cannon, et al., 2018; Tustin, Lamson, et al., 2018) (Table 3). These conditions can impair one's ability to dissipate heat (i.e.,; cool their body) and increase susceptibility for greater heat strain (Dervis et al., 2016; Kenny et al., 2010; Notley et al., 2019; Ribeiro et al., 2004). For example, individuals with type 1 or 2 diabetes have reduced capacity to dissipate heat during exercise (Carter et al., 2014; Kenny et al., 2013; Notley et al., 2019) and demonstrate greater prevalence of heat‐related illness during heat waves (Kenny et al., 2010). Likewise, individuals with hypertension, heart disease and kidney disease may be on medications (e.g., beta‐blockers) that increase their susceptibility to heat intolerance (Epstein & Yanovich, 2019; Pescatello et al., 1987; Puga et al., 2019). Consequently, identification of preexisting medical conditions is encouraged as part of medical monitoring and pre‐placement evaluations (e.g., during the employee onboarding process). In 2011, a Central Texas municipality implemented a heat‐related illness prevention program for outdoor municipal workers that included worker training, acclimatization and medical monitoring. Data from the medical monitoring program revealed of the 604 workers assessed, those with two or more risk factors for heat‐related illness had increased frequency of worker's compensation claims specific to heat‐related illness. After the program was implemented, the number of heat‐related illnesses decreased over the 9‐year study period and the workers' compensation costs also decreased per heat‐related illnesses by an average of 50% (McCarthy et al., 2019).

**Table 3:** Conditions That May Be Associated With Heat Intolerance

Sedentary lifestyle
Type 1 and 2 diabetes
Hypertension
Heart disease
Autonomic dysfunction (dysfunction of the autonomic nervous system that is in control of automatic, unconscious, and involuntary functions of the body)
Kidney disease
Malignant hyperthermia
Medications that affect thermoregulation, central nervous system function, sodium balance
Obesity

It is also prudent to optimize lifestyle behaviors as lack of sleep, poor nutrition, and low fitness have each been individually associated with increased risk of heat‐related illness (Westwood et al., 2021). Table 4 represents a daily heat readiness checklist that workers can use to determine if they have any indications that would increase risk of heat‐related illness. Organizations have implemented worker education that focuses on the mechanism of heat‐related illness and methods to recognize and mitigate common risk factors (e.g., dehydration, sleep deprivation, recent illness, low fitness level) (Riley et al., 2012). However, in many cases where heat‐related illness is reported, failure to implement a heat safety program and lack of compliance with current heat safety guidelines are reported (Nunfam et al., 2018; Tustin, Cannon, et al., 2018; Tustin, Lamson, et al., 2018).

**Table 4:** Recommended Daily Heat Readiness Checklist

**The presence of any of the following indications may place you at greater risk of heat‐related illness**
Dehydration
Lack of sleep
Fatigue or lack of recovery from the previous day
Gastrointestinal discomfort
Not recently eaten or in a fasting state
Psychological stress
**The presence of any of the following indications may place you at greater risk of heat‐related illness and require consultation with medical supervisor before partaking in the work shift**
Signs and symptoms of infection/illness (e.g., common cold, flu, sinusitis)
Fever
Diarrhea
Vomiting
Medications that affect thermoregulation, central nervous system function, sodium balance (e.g., beta‐blockers)

#### 4.1.3 Gaps in Knowledge

1. Further understanding the relative contribution of identified risk factors (e.g., dehydration, disease status, medication, age, body composition, environmental condition, fitness level) and how they influence worker tolerance to heat stress in the occupational space is required.
2. Establishing ways to identify and protect the privacy of workers who are characterized as “high risk” using limited resources, in a cost‐effective way is essential.
3. Strategies for effective implementation of behavioral changes interventions in workers and the organization toward better heat hygiene practices.

### 4.2 Hydration

#### 4.2.1 Background and Significance

Maintaining an adequate level of hydration and electrolyte balance is important for optimizing human health and both physical and cognitive performance, particularly in extreme environmental conditions (Cheuvront & Kenefick, 2014). The following section will discuss the current literature surrounding the influence of hydration on worker health and considerations related to individual fluid needs and fluid access and availability as it pertains to the workplace.

#### 4.2.2 Current Research

##### 4.2.2.1 Hydration for Worker Health

Regulation of total body water is a complex and dynamic process. For the purposes of this discussion, the following definitions have been used: *euhydration* will refer to normal body water content; *hypohydration* will refer to the steady‐state of a total body water deficit; *hyperhydration* will refer to the steady‐state of a total body water excess; *dehydration* will refer to the process by which body water is lost within the body (e.g., sweating, urine and fecal losses, respiration); *rehydration* will refer to the process by which body water is restored within the body; *underhydration* will refer to a state of normal body water that is associated with decreased water intake, increased urine osmolality, and increased secretion of arginine vasopressin (also known as antidiuretic hormone) (Greenleaf, 1992; Kavouras, 2019).

Hydration can have important short‐ and long‐term impacts on worker health. For instance, overdrinking can increase the risk of hyponatremia (i.e., abnormally low levels of sodium in the blood) if a volume of hypotonic solution (e.g., low concentration of solutes) such as water is consumed so rapidly that the volume is not removed from the circulation by the kidneys before dramatically reducing circulating sodium concentration. Although plausible, hyponatremia is relatively rare in the workplace. In contrast, underhydration is quite common, particularly when environmental temperature is elevated (Piil et al., 2018). A hypohydrated state may impact work and health outcomes, and more recent findings support a role for repeated exposure to a hypohydrated state in deleterious health outcomes (Lucas et al., 2013; Mansor et al., 2019; Schlader et al., 2015). When exposed to environmental heat, hypohydration leads to reductions in physical work capacity and productivity (Cheuvront & Kenefick, 2014; NIOSH, 2016), increased risk of heat‐related illness (Lucas et al., 2013; Mansor et al., 2019; Schlader et al., 2015), reduced cognitive function and alertness, as well as, fatigue (Adan, 2012; Ganio et al., 2011). All of these outcomes can undermine health and safety in the workplace. A meta‐analysis of 14 studies examining the physiological and productivity effects of occupational heat stress reported that working a single shift in the heat resulted in a 14.5% increase in urine specific gravity, a marker of dehydration, in workers compared to those working a shift in a thermoneutral condition (e.g., no heat) (Flouris et al., 2018).

More recently, the impact of hypohydration on aspects of worker health beyond the workplace has begun to be elucidated. For instance, repeated exposure to a hypohydrated state caused by severe physical work in the heat has been proposed to bring about chronic kidney disease, which is speculated to be due to the workers experiencing repeated bouts of subclinical kidney injury (Glaser et al., 2016; Hansson et al., 2020; Johnson et al., 2019; Mix et al., 2018; Nerbass et al., 2017, 2019; X. Yang et al., 2020). Cases of chronic kidney disease have been reported in workers performing manual work in hot environments in hottest regions in the world (Aguilar & Madero, 2019; Butler‐Dawson et al., 2019; Glaser et al., 2016; X. Yang et al., 2020). Interestingly, these cases have occurred in the absence of its classic causes of chronic kidney disease, which suggests a potential occupational etiology (Johnson et al., 2019). The proposed mechanism for kidney injury (acute and/or chronic kidney disease) in agriculture workers stems from kidney dysfunction associated with the combined effects of direct toxicity (pesticides, heavy metals, etc.), occupational heat stress, and dehydration (Tucker et al., 2017). A recent study by Butler‐Dawson and coworkers, found that dehydration measured by increased urine specific gravity was associated with a greater incidence of acute kidney injury.

##### 4.2.2.2 Individual Fluid Needs

To maintain adequate hydration, an individualized approach to developing hydration strategies is warranted. The volume of fluids needed to maintain adequate hydration varies person‐to‐person and is dictated by factors such as the environmental conditions, individual sweat rate, exercise intensity, sex, and required protective equipment (Baker & Jeukendrup, 2014). In occupational settings, evidence suggests that the prevalence of hypohydration before, during and after the work shift is high (Biggs et al., 2011; Brake & Bates, 2003; Kenefick & Sawka, 2007; Piil et al., 2018), highlighting the importance of targeted approaches to optimize hydration practices in this space.

The approach to optimizing hydration in occupational settings should focus on pre‐shift, during‐shift, and post‐shift time points. Employers cannot dictate fluid consumption before and after the work, but employers should encourage workers to arrive to their shifts in a euhydrated state. This is important with evidence showing that 40%–70% of workers arrive to their shifts hypohydrated (Biggs et al., 2011; Brake & Bates, 2003; Piil et al., 2018). During work shifts, promoting fluid consumption to minimize fluid losses is essential to mitigate dehydration‐related reductions in performance/productivity (Piil et al., 2018). Designing work‐to‐rest ratios based on environmental conditions, intensity/workload, and required protective clothing, allows for workers to minimize fluid losses to offset hypohydration, and provide them with opportunities to replace fluid losses due to sweating during their working shifts (Brake & Bates, 2003; Kenefick & Sawka, 2007; Trites et al., 1993). Following a shift, workers should be encouraged to consume fluids to replace remaining water losses from sweat. When coupled with hydration education for workers who are new and/or who experience high heat exposure, assessing pre‐ and post‐shift body weight changes, urine color, and sensation of thirst are helpful strategies to guide individuals need a day‐to‐day basis (Cheuvront & Kenefick, 2016).

Beverage composition is an important factor to consider for promoting hydration in occupational work. For prolonged work, particularly in hot environmental conditions, consuming fluids containing carbohydrates and electrolytes may improve overall fluid consumption due to the increased palatability (Clapp et al., 1999). For example, Clapp and coworkers, found that occupational workers consumed a greater volume of fluids and exhibited a lower body mass loss when consuming fluids containing 6% carbohydrates and either 18 or 36 mEq/L of sodium during work in a hot environment (Clapp et al., 2000). However, water (diluted carbohydrate‐electrolyte solutions can also be considered) should be the preferred fluid that is consumed due to the long‐term health implications on added energy intake (Miller & Bates, 2010). Consideration of cultural alternatives for beverages with electrolytes should be included in hydration promotion programs (i.e., coconut water). In addition, access to cool beverages will also increase the volume consumed over a given period of time (Clapp et al., 1999), which should be taken into consideration when designing hydration strategies in occupational settings. Clapp and coworkers found that the use of a carbohydrate electrolyte beverage (i.e., 6% carbohydrate) that was maintained at approximately 18°C was effective at minimizing fluid losses in occupational workers exposed to heat stress (Clapp et al., 2000).

##### 4.2.2.3 Behavioral Aspects Guiding Fluid Consumption

Health behaviors related to fluid consumption vary across the population with evidence indicating that many adults are inadequately hydrated (Mekonnen & Hoekstra, 2016; Miller & Bates, 2010; UNICEF, 2020, “*Progress on drinking water, sanitation and hygiene in schools | UNICEF*, https://www.unicef.org/reports/progress-on-drinking-water-sanitation-and-hygiene-in-schools-focus-on-covid-19; WHO, 2019). Further, differences in habitual fluid intake have been observed between sex (Mekonnen & Hoekstra, 2016) and race/ethnicity (Bethancourt et al., 2021; Miller & Bates, 2010; Rosinger, 2018; Venugopal et al., 2016). It must be noted that fluid intake behaviors are driven by a number of factors including cultural beliefs, knowledge of hydration on health, access to safe and affordable sources of drinking fluids, and trust/distrust of water sources (Bethancourt et al., 2021; Miller & Bates, 2010; Venugopal et al., 2016). Developing effective and tailored educational programs surrounding healthy hydration (i.e., adequate water intake and reduced consumption of sugar‐sweetened beverages) and the associated benefits related to health and performance for workers may encourage an environment that supports proper hydration in these populations.

##### 4.2.2.4 Worksite Considerations

Access and availability of fluids is of particular concern with regards to hydration considerations in occupational settings. Specifically, the scarcity of fresh groundwater and access to clean drinking water in certain geographic areas (Mekonnen & Hoekstra, 2016), and the concern over contaminated water sources (UNICEF, 2020, https://www.unicef.org/reports/progress-on-drinking-water-sanitation-and-hygiene-in-schools-focus-on-covid-19; WHO, 2019) supports the hypothesis of water insecurity being associated with the risk of underhydration, particularly in persons subjected to heat stress (Bethancourt et al., 2021; Rosinger, 2018).

When developing evidence‐based hydration strategies, it is important to consider the specific work settings where individuals perform work. In remote settings that have little to no access to clean drinking water, extensive planning involving the acquisition, delivery, and placement of clean drinking water is needed to ensure unlimited access to fluids by all workers. Regulation and enforcement mechanisms also need to be implemented to ensure that the water provided to workers meets clean water standards. In settings where drinking water is more readily available, efforts for installing an adequate number of drinking stations or having a centralized location (e.g., breakroom) where workers can rehydrate allows for the promotion of fluid consumption.

Facility design can potentially be an important factor surrounding hydration‐related issues in occupational settings. Having access to a clean bathroom may influence one's desire to consume fluids during their working shift. A recent study (Venugopal et al., 2016) found that increased heat exposure was associated with greater sweat losses and that unsanitary facilities or inadequate/no access to a toilet, increased the risk of reported genitourinary complaints. It is also common for individuals with minimal access to bathrooms (e.g., agricultural workers) to voluntarily restrict their fluid intake to avoid the urge to urinate. In addition, as described above, access to clean drinking water is vital to promote proper hydration during working hours. While the implementation of these considerations may differ depending on occupational sector (e.g., portable bathrooms in outdoor construction/agricultural locations and clean water jugs vs. drinking water sources and clean bathrooms that are proximal to one's working site in industrial/manufacturing settings), it is crucial that supervisors/managers/foremen provide these resources to workers.

#### 4.2.3 Gaps in Knowledge

1. Determine occupation‐specific mechanisms associated with the impact of dehydration on heat‐related illness risk, productivity and health and safety in workers exposed to environmental heat stress.
2. Understanding the impact of hypohydration, with or without heat stress on occupational health and well‐being in the workplace.
3. Understanding how physical, social, and environmental factors that are associated with fluid intake and the development of hypohydration on both the micro‐ (days) and macro‐timescales (weeks, months, years) impact health and performance outcomes.
4. Understanding the ramifications of piece‐pay structures (e.g., paid per bundle harvested) on hydration.

### 4.3 Heat Acclimatization

#### 4.3.1 Background and Significance

For occupational workers exposed to hot environmental conditions, both outdoors and indoors, HA is an effective strategy to reduce the risk of heat‐related illness in the workplace (NIOSH, 2016). HA is defined as repeated bouts of physical activity in a hot environment that induce physiological adaptations that reduce strain and improve thermal tolerance during physical activity (Périard et al., 2015). These physiological adaptations enhance sudomotor (i.e., earlier onset of sweating, greater sweat production, increased sweating efficiency and reduced electrolyte loss in sweat), thermoregulatory (i.e., lower work internal temperature), cardiovascular (i.e., lower work heart rate, increased skin blood flow at a given core temperature, expanded plasma volume) function, and worker productivity (ACGIH, 2017; Armstrong & Maresh, 1991; Moseley, 1994; NIOSH, 2016). Without continued heat exposure following the initial HA period, most adaptations from HA are lost (i.e., decay) within 3 weeks (Daanen et al., 2018). Heat re‐acclimatization (RHA) has been proposed as a method to overcome decay, since it simply requires that the HA process be repeated over 4–7 days (Daanen et al., 2018). Another method to mitigate HA decay is to experience heat exposure once every fifth day to maintain the initial HA adaptations (J. L. Pryor, Pryor, Vandermark, Adams, VanScoy, et al., 2019; R. R. Pryor, Pryor, Vandermark, Adams, Brodeur, et al., 2019).

The physiological adaptations associated with a HA program are shown to reduce the risk of heat‐related illness (Park et al., 2017), reduce physiological strain (Moseley, 1994; Périard et al., 2015), and improve physical performance (Benjamin et al., 2019). Employers benefit from implementing HA programs because the physiological adaptations can improve or maintain labor productivity and work capacity (Kjellstrom et al., 2016). A properly designed HA plan will utilize the initial week of employment for new workers or workers returning to work after a prolonged absence to gradually expose workers to the heat and/or workload of a full shift.

The importance of HA is well recognized in the scientific and medical communities, yet the practice of implementing a HA plan in occupational settings continues to be an abstract and often a neglected element of the workplace heat illness prevention programs. Neglecting HA programs is particularly true for smaller businesses that may be lacking occupational safety and health resources such as a professional full‐time safety manager (Jacklitsch et al., 2018; Sinclair & Cunningham, 2014). Understanding the current research, highlighting best practices, and identifying gaps in knowledge are important to continue the discussion on how to successfully implement HA plans at a wide variety of workplaces that may vary in levels of knowledge and resources.

#### 4.3.2 Current Research

The practice of HA in workers gained traction in the mid‐20th century upon observation that miners who were acclimatized to the extreme heat conditions of mining experienced less physiological strain and fewer symptoms of heat‐related illness than their unacclimatized peers (Weiner, 1950). In other occupational settings including the military, heat‐related illness is commonly observed in individuals who are not heat acclimatized (Park et al., 2017). Since HA reduces thermoregulatory, cardiovascular, and metabolic strain while improving work tolerance (Périard et al., 2015), implementing this strategy in occupational settings that expose workers to thermally stressful environments is useful for reducing heat‐related illness incidence while improving worker safety and productivity.

Worker responses to heat stress vary throughout the HA process, providing important information regarding the implementation of this strategy in workers. The second consecutive working day in the heat results in increased fatigue, core body temperature, and symptoms of heat‐related illness compared to the first day (J. L. Pryor, Pryor, Vandermark, Adams, VanScoy, et al., 2019; R. R. Pryor, Pryor, Vandermark, Adams, Brodeur, et al., 2019; Schlader et al., 2017). The progression of HA has been shown to reduce heat strain over a 5–14 day period depending on the HA protocol (Armstrong & Maresh, 1991). Therefore, adhering to recommendations such as implementing work‐to‐rest ratios and adequate hydration is particularly important to ensure safety during the first few days of HA (NIOSH, 2016). Of note, research has shown that maintaining hydration optimizes the HA process (Sekiguchi et al., 2020; Travers et al., 2016).

**Figure 1:**
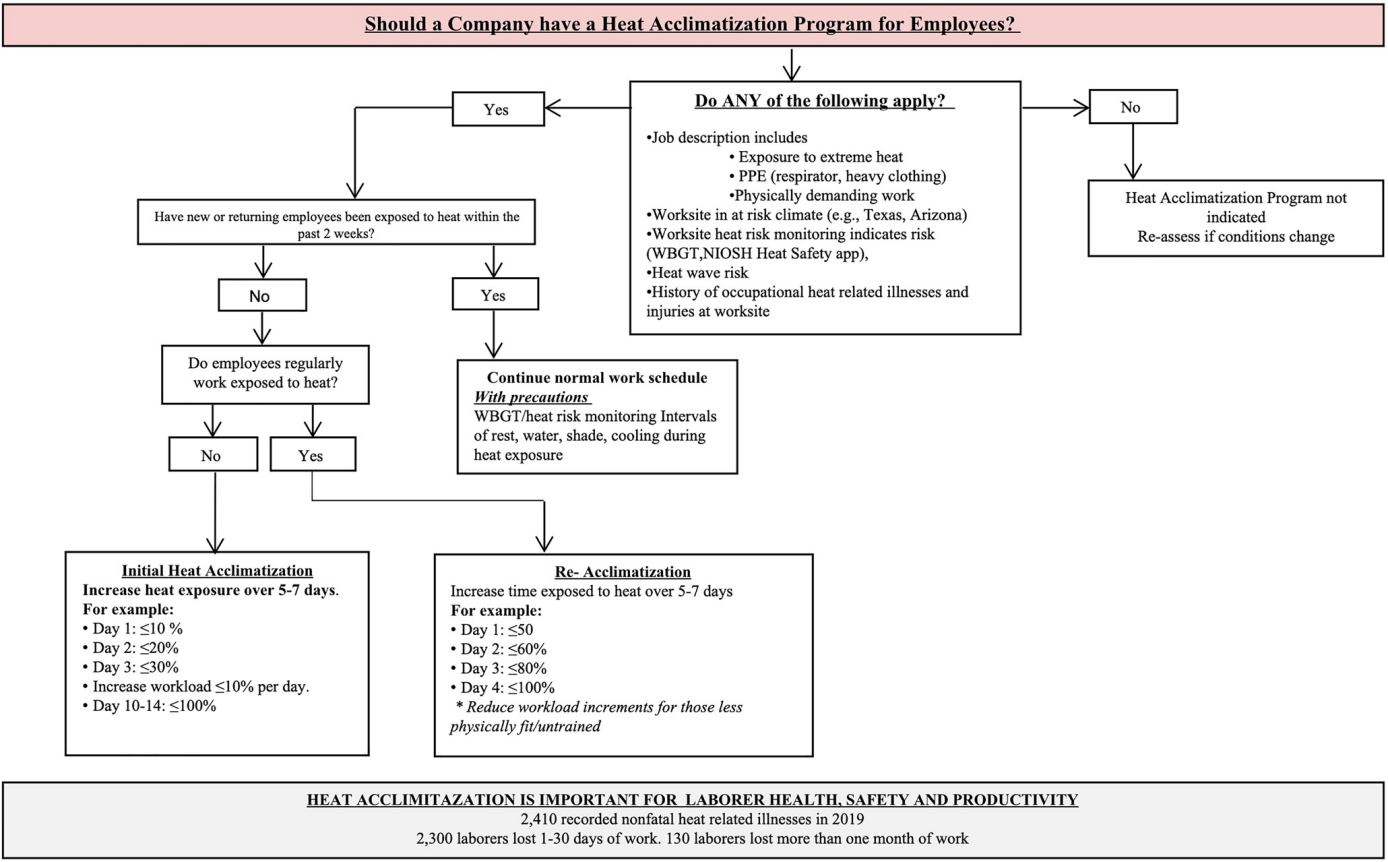
Occupational heat acclimatization and safety guidelines. NIOSH, National Institute of Occupational Safety; PPE, personal protective equipment; WBGT, wet bulb globe temperature.

Despite this knowledge of best practices, recent studies investigating risk factors for heat‐related illness in workers reported that the majority of heat‐related fatalities occurred during the first week of work (Arbury et al., 2016; Tustin, Cannon, et al., 2018; Tustin, Lamson, et al., 2018) and at worksites where the employers did not impose a HA policy (Tustin, Cannon, et al., 2018; Tustin, Lamson, et al., 2018). Research regarding the practical implementation of HA in workers is lacking, with current best recommendations outlining the gradual increase in exposure time across the first 1–2 weeks, with a more conservative approach for workers who are new to the job (NIOSH, 2016). Figure 1 presents an example of an algorithm that employers can follow to initiate HA.

#### 4.3.3 Gaps in Knowledge

1. Investigate how HA protocols can be best applied while maintaining productivity.
2. Job‐specific HA protocol must be created due to generalized and nonspecific HA guidelines from governing bodies and due to a wide variety of physical demands seen in the occupations at risk of heat‐related illness.
3. Quantification of intensity and duration for HA protocol (i.e., calculating metabolic rate, workload) across all occupations.
4. Minimum acceptable fitness level (estimated VO2max) for each occupation prior to beginning work in the heat (e.g., HA).
5. Thermoregulatory and cardiovascular adaptations to HA programs in diseased working populations (e.g., diabetic, hypertensive).

### 4.4 Environmental Monitoring

#### 4.4.1 Background and Significance

It is well established that the ambient environment contributes to the risk of heat‐related illness (Spector et al., 2019). Environmental monitoring is therefore a key component of heat safety. By accurately and continuously monitoring the environmental conditions experienced by workers, employers can implement effective interventions to mitigate heat‐related illnesses, while not over protecting, which may result in a reduction in productivity.

#### 4.4.2 Current Research

##### 4.4.2.1 Ambient Environmental Conditions and Heat Exposure Assessment

Accurate and localized measurements of the meteorological variables defining human heat stress are critical for heat‐health risk management (Hosokawa et al., 2019). These variables include air temperature, air speed, relative humidity, and radiant heat (e.g., solar radiation in outdoor settings).

There are various heat stress indices that integrate various meteorological variables such as the wet bulb globe temperature (WBGT), and the heat index (Table 4). The WBGT is commonly used for occupational health and decision‐making (ACGIH, 2017; Budd, 2008; ISO 7423, 2017; NIOSH, 2016). Outdoors, WBGT is defined by a weighted sum of the natural wet bulb temperature (0.7*T*_nwb_), black globe temperature (0.2*T*_g_), and shaded air temperature (0.1*T*_a_). An indoors variation of this index is computed as the weighted sum of *T*_nwb_ (0.7*T*_nwb_) and *T*_a_ (0.3*T*_a_). The heat index approximates a human heat balance model that uses inputs of temperature and relative humidity, and is widely available (Rothfusz, 1990). Heat index can be used with the understanding that adjustments for sun exposure (or radiant heat in general), metabolic demands, and clothing are needed (e.g., Bernard & Iheanacho, 2015). Further, in certain hot and dry locations, air temperature alone is more appropriate to use than a heat index in determining necessary interventions to prevent heat‐related illness (Anderson et al., 2013).

Metrics such as WBGT can be measured with portable meteorological sensors or via models with meteorological data inputs (Table 5). On‐site measurements best capture local conditions, but accuracy can vary among portable sensors, influencing activity modification thresholds (e.g., Cooper et al., 2017). If direct measurements are not available, modeled WBGTs or other heat metrics from representative weather measurements (e.g., online calculators) can be a suitable alternative, although the accuracy of modeled values like WBGT can vary greatly based on inputs and model assumptions (Grundstein & Cooper, 2020; Lemke & Kjellstrom 2012; Liljegren et al., 2008). Online calculators and apps such as OSHA outdoor WBGT calculator (osha.gov), are available that can estimate heat stress metrics like WBGT and heat index with inputs of location and weather data (“*Heat—OSHA outdoor WBGT calculator—Occupational Safety and Health Administration*,” OSHA, n.d.). Weather forecast products can also help with heat safety planning (NOAA, n.d., www.graphical.weather.gov).

**Table 5:** Considerations in Monitoring Environmental Conditions for Occupational Heat‐Hazard Assessments

Monitoring weather variables		Advantages	Disadvantages	Adjustments
Location	On‐site with portable weather sensor at 1.1 m height	Best represents workers' environmental conditions; provides accurate classification of heat exposure	Cost of portable sensor, maintenance, ease of use	
	Off‐site weather station observations or model output	Low‐cost/free, ease of use via apps	May not be representative of local conditions, leading to misclassification of heat exposure	Interpolate values from 2 or 3 weather stations
Indices calculated from environmental measures	WBGT industry standard	Combines multiple meteorological variables for a more comprehensive heat stress measure	Monitoring equipment costs; lower‐cost equipment may be less accurate	Must account for clothing adjustment factor; acclimatization; metabolic load
Indices calculated from heat balance models	Heat index^a^	Simple to determine; widely available; widely used unit; broadly known	Solar, clothing, and activity assumptions not representative of most working conditions; does not work in very dry climates (avoid use)	Add solar factor and adjustments for metabolic rate and clothing
	UTCI	Publicly available version (regressions) simple to determine, widely used unit (°C). Accounts for the full environment	Built to assess thermal stress in average person; not developed for working population; does not yet have adjustments for metabolic rate	Clothing is adapted based on air temperature (0.30–2.6clo range)
	PET	Publicly available software easy to use, widely used unit (°C). Accounts for the full environment. Use mPET if making calculations for workers	Built to assess thermal comfort for an average person; assumes “light activity” and that one is not moving with constant clothing (0.9clo). Cannot modify clothing or METs	

Abbreviations: MET, metabolic equivalent of task; mPET, modified physiological equivalent temperature; PET, physiological equivalent temperature; UTCI, universal thermal climate index; Apps, applications; WBGT, wet bulb globe temperature.

^a^ Basic rational index simplified from its original version (apparent temperature) and derived from only air temperature and humidity in its current form.

##### 4.4.2.2 Accounting for Nonenvironmental Factors in Heat Stress Exposure Assessment

A full heat stress exposure assessment in occupational settings considers environmental conditions, metabolic demands, and clothing requirements in conjunction with an individual's acclimatization state. The universal thermal climate index (UTCI) and physiological equivalent temperature (PET) are two thermal indices that account for metabolic and physiological demands to obtain a better assessment of heat strain in workers (Błażejczyk et al., 2013; Höppe, 1999). UTCI is a human model that predicts thermoregulatory responses involved in heat balance under different environmental conditions (Błażejczyk et al., 2013). Similarly, PET uses an energy balance model to predict thermoregulatory responses (Höppe, 1999). The goal of occupational exposure limits to heat stress is founded on the premise of a sustainable heat stress exposure during which core temperature demonstrates stability below a critical threshold of 38–39°C depending on the literature (ACGIH, 2017; NIOSH, 2016). As more research emerges on core temperature responses of workers in the heat across various occupations, the critical threshold may need to be re‐evaluated.

**Figure 2:**
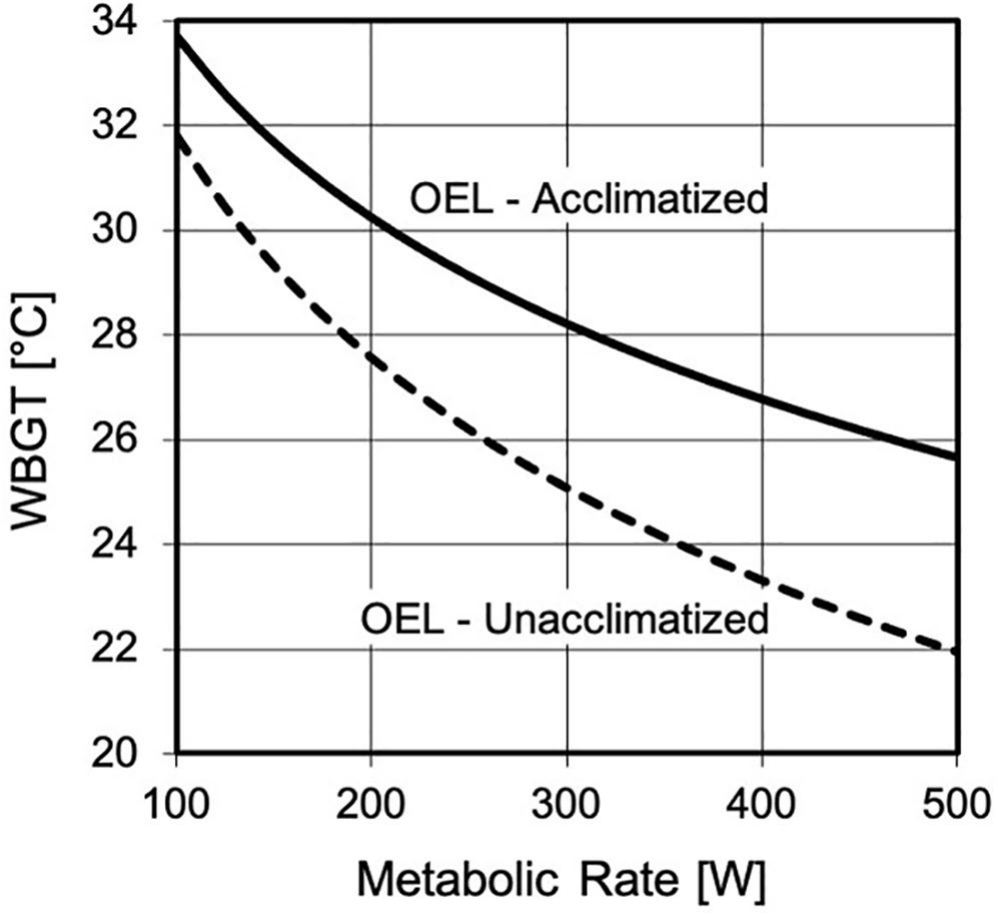
Occupational exposure limit (OEL) as a limiting wet‐bulb globe temperature (WBGT) at a given metabolic rate for heat acclimatized and nonheat acclimatized individuals. Adapted from ACGIH (2017).

There are multiple approaches to establishing safe heat exposure limits. Environment, work demands, and clothing are recognized risk factors; however, the duration of exposure is also a critical factor (ACGIH, 2017; NIOSH, 2016). Limits based on WBGT or heat index are usually based on sustained exposures for long periods. If the exposures are planned for short durations, then there are alternative methods for heat stress assessment (e.g., U.S. Navy Physiological Heat Exposure Limit, Predicted Heat Strain [ISO 7933]) (Bernard et al., 2005; ISO 7933, 2004). At present, WBGT is the most frequently used to represent the environmental conditions across a workday, although many other direct, rational (i.e., indices based on calculations using the heat balance equation), and empirical heat stress indices are available (NIOSH, 2016). To account for metabolic heat generation, the threshold WBGT is adjusted to match an estimated metabolic rate (Figure 2).

OELs generally assume healthy individuals wearing ordinary work uniforms. Other clothing ensembles can change the maximum rate of evaporative cooling from that of the reference clothing (Bernard et al., 2005, 2008). To account for the differences, WBGT‐based clothing adjustment values (CAV) have been proposed to account for the clothing differences so that the effective WBGT of the exposure is the ambient WBGT plus the CAV (ACGIH, 2017). The occupational heat stress limits can be adjusted to account for HA state by providing an OEL for a non‐HA person.

#### 4.4.3 Gaps in Knowledge

1. Understanding off‐site data and models to estimate on‐site exposures.
2. The link between OELs or other metrics with health effects or other occupational heat stress outcomes (e.g., productivity, errors, quality) remain unknown.
3. Intervention thresholds for shorter (<1 h) versus longer heat exposures (>1 h) and whether they vary based on worker characteristics (e.g., age, body mass index).

### 4.5 Physiological Monitoring

#### 4.5.1 Background and Significance

Quantifying thermal strain during work in a hot or humid environment typically relies on information about the environment, clothing, and workers' metabolic rate (ACGIH, 2017; NIOSH, 2016). Although this approach is encouraged, it assumes that workers are physiologically homogenous and have similar levels of fitness, acclimation statuses, behavioral strategies, and other individual characteristics. To account for individual factors to improve safety and performance during work in the heat, wearable physiological status monitoring has been proposed in the occupational setting. Physiological monitoring of vital signs (e.g., heart rate, body temperature) collects the worker's individual response to exertion and environmental conditions in real‐time and may offer a greater level of protection from heat‐related injury compared to self‐monitoring.

#### 4.5.2 Current Research

Despite the growing use of physiological monitoring for heat‐related illness in athletic and military settings (Davison et al., 2009; Friedl, 2018; Kiely et al., 2019), research is limited in the occupational setting (i.e., labor force). In occupational workers, the utilization of valid and reliable physiological monitoring devices is limited to research where direct measures of physiological responses such as ingestible gastrointestinal temperature capsules and heart rate monitoring are feasible (Notley et al., 2018). Although these measures are considered valid and appropriate to quantify thermal strain, the equipment is costly and/or single use disposable (i.e., no chronic measures), limiting feasibility in many occupational settings (Notley et al., 2018). In addition to direct measurements, multiple models of predicting thermoregulatory responses have been proposed and have varying degrees of success in different environments (Buller et al., 2013; Frank et al., 2001; Moran et al., 1998; Pandolf & Goldman, 1978). To predict thermal strain, however, these models require either a direct measurement, or an accurate estimation of core body temperature.

The field of wearable physiological sensors and technologies is rapidly growing. Current wearable technologies have been developed to be worn under clothing or on the wrist and can measure a variety of parameters including heart rate, skin temperature, and activity in real time (Brearley et al., 2015; Cuddy & Ruby, 2011; Hunt et al., 2016); these measures can also estimate additional physiological responses such as body temperature using algorithms. Although wearable sensors and technologies provide a valuable opportunity to evaluate thermal strain of workers in the heat without interrupting standard working procedures, many of these devices have not been validated in occupational settings and their efficacy to alter safety guideline decisions and conduct medical surveillance remains unknown (Bourlai et al., 2012; Holm, et al., 2016).

Implementation of physiological monitoring devices is also challenging as the data presented by a physiological monitoring device must be easily interpreted and actionable by the worker or a designated medical monitor. There is considerable variation in an individual's ability to tolerate thermal strain so it is unlikely that a single estimated physiological parameter will signal impending morbidity in all workers. Lastly, there must be a willingness by the end user to act on the information, which will require the cooperation of both workers and management. Employers and safety managers are encouraged to follow the development and deployment of valid and reliable (within their given worksite) physiological monitoring systems in the occupational setting for future use and to consider their adoption when these devices provide information that will help limit risk of heat‐related illness.

#### 4.5.3 Gaps in Knowledge

1. The validity and reliability of various wearable sensors and technologies in different occupational settings.
2. Strategies to effectively implement validated physiological monitoring systems during occupational work.
3. The critical thresholds of various physiological parameters for risk stratification and management.

### 4.6 Body Cooling

#### 4.6.1 Background and Significance

Body cooling is an effective, albeit underutilized heat management strategy to reduce thermal strain, prevent heat‐related illness, and improve work productivity (Foster et al., 2020). OSHA's heat illness prevention campaign, “Water.Rest.Shade” encourages employers to provide workers with a cool location to rest and recover from heat exposure. Many investigations (Casa et al., 2015; McEntire et al., 2013) suggest that short periods of passive rest have little effect on physiological recovery (i.e., reduction in core temperature and heart rate), particularly during repeated bouts of physically demanding work in the heat. Moreover, OSHA's recommendation for “shade” is limited to outdoor workers exposed to the sun and does not include indoor workers experiencing heat (“*OSHA's campaign to prevent heat illness in outdoor workers | heat fatalities [text version] | Occupational Safety and Health Administration*,” OSHA, 2011). Therefore, cooling modalities (i.e., garments or other body cooling modalities) and strategies to limit heat strain can be implemented with the intent to preserve and improve physical and cognitive performance, and enhance worker health, safety and productivity (Chicas et al., 2020; DeMartini et al., 2011; McDermott et al., 2009). This section focuses on considerations for implementing effective body cooling modalities based on the employers' worksite.

#### 4.6.2 Current Research

**Figure 3:**
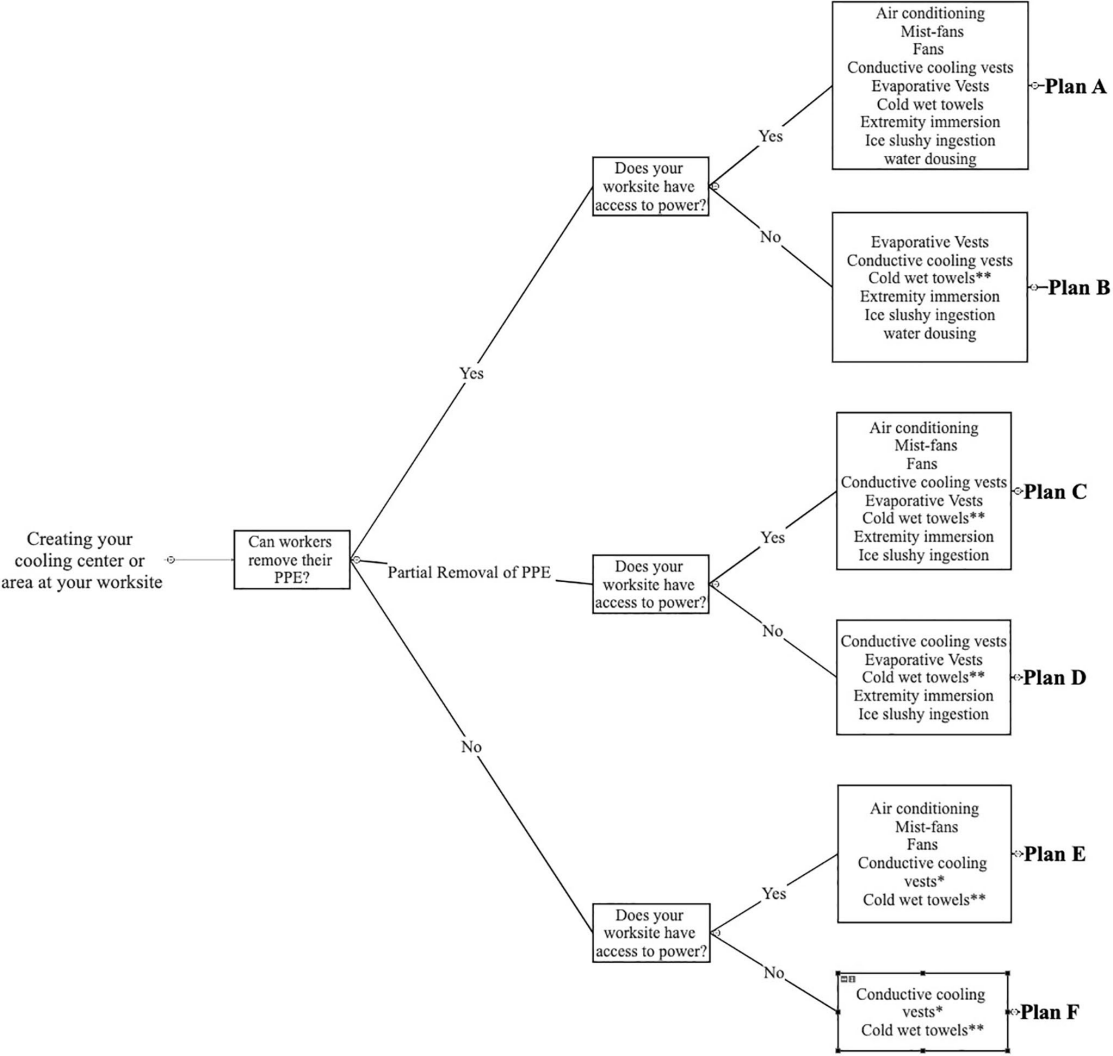
Cooling modalities to use for cooling center based on resources. Note that *must be donned prior to work shift; **cold wet towels must be rotated every 1–2 min to obtain optimal cooling potential; PPE, personal protective equipment.

The effectiveness of body cooling interventions used in the occupational setting is dependent on the resources available on the worksite, environmental conditions, personal protective gear requirements, shift organization and duration, occupation, and many other factors (Chicas et al., 2020). Table 6 presents active cooling strategies to mitigate thermal strain (Butts, et al., 2017; Casa et al., 2007; Chicas et al., 2020; DeMartini et al., 2011; Hospers et al., 2020; McDermott et al., 2009; Morris et al., 2016). It is important to note that whole‐body cold‐water immersion produces the most effective cooling rates; however, it lacks feasibility for implementation at the worksite (Casa et al., 2007). This table also highlights cooling rates, estimated cost, requirements for implementation, and the benefits and limitations of each proposed cooling method within the occupational setting. Physiological effects of cooling strategies (cooling rate, change in core temperature) are often accompanied by increases in perceptual measures (i.e., thermal comfort), improved health status, improved cognitive performance, and enhanced productivity (Cheung, 2010; Kjellstrom et al., 2016; Parsons, 2009; Song & Wang, 2016; H. Yang et al., 2019; Zhao et al., 2015). For example, one of the most effective methods to maintain productivity is to improve thermal comfort of workers (Gunn & Budd, 1995; Kjellstrom et al., 2016). Figure 3 and Table 7 also provide a flowchart and equipment list (respectively) to assist supervisors and employers to create a heat safety plan which will aide in protecting their employees, but importantly, in maintaining productivity levels by utilizing body cooling strategies.

**Table 6:** Active Cooling Strategies With Corresponding Benefits and Limitations

Active cooling strategy	Cooling effectiveness	Cost estimates	Requirements for implementation^a^	Benefits in occupational setting	Limitations in occupational setting
Whole body ice and/or water immersion	High	100 gal: $90–170	Accessibility to a water source, a large immersion tub, ice	Considered the gold standard for EHS treatment	Not accessible in remote settings
				Employers should have an immersion tub on site for EHS cases	May require removal of PPE and layers of clothing for *nonmedical emergencies*
		150 gal: $160–200		Strongly supported by scientific evidence	Unlikely to implement during rest breaks for nonmedical emergencies
					Employers are unlikely to provide each worker with their own immersion tub for nonmedical emergencies
Extremity immersion	Low‐med	$150–2,000	Accessibility to a water source or ability to transport coolers for immersion, ice	Allow workers to keep their PPE on during cooling	Requires cold water temperature (5°C) to elicit higher cooling rates
				Can use water coolers to mimic forearm immersion troughs	Not effective in rest periods that would occur in occupational setting (<30 min)
					Little research on effects on hand dexterity
Hand cooling	Low	$30–120	Accessibility to a water source or ability to transport coolers for immersion, ice	Allow workers to keep their PPE on during cooling	Minimal surface area being cooled, less effective
				Easy to provide to individual workers	Little research on effects on hand dexterity
Air‐conditioning	High	$3,000–13,000	An air‐conditioned room	Able to remove the environmental heat stress completely	Economically and environmentally costly
				Strongly supported by literature	Cannot implement during work for outdoor workers
				Does not require the removal of PPE	Not personalized
Air movement (ventilation, electric fan, mist‐fan)	Med	$10–10,000	An electric fan, power source	Effective in hot, humid conditions, which represents most heat wave conditions	Can be detrimental in hot, very dry conditions
				Can become personalized	Not effective if workers are wearing heavy PPE
				Can be transported	
				Lower cost compared to air‐conditioning	Use is limited to 1–3 workers (dependent on size of fan)
				Increases evaporative potential and supported in the literature	
Head cooling	Low	$3–300	Head cooling device (towel, cap, etc.)	Can be used under helmets or work hats during shifts	Little support from scientific literature
				Low cost	
				Easy to implement and provide to all workers	Covers small amount of body surface area
				Does not require the removal of PPE	
Cold, wet towels	Low	$10–50	Coolers for storage if required	Low cost	Must keep towels cold and rotate often
					Does not cover the whole body
				Does not require full removal of PPE	Difficult to use under PPE
					Towels require preparation
Conductive cooling vests (phase change, ice)	Med	$30–3,000	Vest and replaceable ice pack/coolant	Effective in any environmental condition	Economically and environmentally costly
				Can be worn underneath PPE and used during work	Coolant or ice can melt
			Some require tubes within garment with cooling refrigerant source	Can be used in remote settings	Requires worker to “carry” extra load from coolant
				Supported in scientific literature	Employers must provide a cooling vest to each worker
Evaporative cooling vests	Med	$30–3,000	Evaporative vest	Effective in hot, low humidity conditions	Less effective in high humidity or under PPE
				Facilitates air flow with the fabric of the vest	Employers must provide a cooling vest to each worker
				Can be used in remote settings	Limited research in remote occupational settings
				Less expensive than conductive cooling vests	
Water dousing	Low	$1.50–20	Water bottle or hose	Few supplies needed	Requires removal of PPE
				Easy to implement	Can cause discomfort with wet garments if PPE not removed
				Low cost	Limited research on effects of water dousing in occupational setting
Ice slushy ingestion	Low	$1–10	Water, ice, cooler for storage	Low cost	Must be able to keep beverage cold
				Easy to implement	
				Does not require full removal of PPE	May cause reduction in sweating response, should be implemented at rest
				Helps with hydration	

*Note*. Cooling effectiveness: high, >0.155°C/min; med, 0.078–0.154°C/min; low, <0.078°C based on McDermott et al. (2009). While some cooling modalities do not require the removal of PPE, PPE should be removed whenever possible in order to maximize cooling.

Abbreviations: EHS, exertional heat stroke; PPE, personal protective equipment.

^a^ Requirements are dependent on specific work setting and resources.

**Table 7:** Equipment List for Cooling Center (Figure 3)

**Plan A (access to power and full PPE** **removal)**
Mist‐fan, fan, cooling vests, cold wet towels, ice, water
Refrigerator (any size) or coolers for storage of ice, cold water, cold wet towels, for cooling vest insertions
Water bottles or cups for hydration or water dousing (storage in the cold)
Water spigot and hose to fill immersion tub
Plastic tub for extremity immersion
Nearby power outlet and extension cords for mist‐fans, fans, refrigerators
**Plan B (no access to power and full PPE removal)**
Cooling vests, towels, ice, water
Coolers for storage of ice, cold water, cold wet towels, for cooling vest insertions
Water bottles or cups for hydration or water dousing (storage in the cooler)
**Plan C (access to power and partial PPE removal)**
Mist‐fan, fan, cooling vests, cold wet towels, ice, water
Refrigerator (any size) or coolers for storage of ice, cold water, cold wet towels, for cooling vest insertions
Water bottles or cups for hydration (storage in the cold) if applicable
Water spigot and hose to fill immersion tub
Plastic tub for extremity immersion (i.e., forearm, hand)
Nearby power outlet and extension cords for mist‐fans, fans, refrigerators
**Plan D (no access to power and partial PPE removal)**
Cooling vests, towels, ice, water
Coolers for storage of ice, cold water, cold wet towels, for cooling vest insertions
Water bottles or cups for hydration (storage in the cooler)
Plastic tub for extremity immersion (i.e., forearm, hand)
Dry towels before and after immersion
**Plan E (access to power and no PPE removal)**
Mist‐fan, fan, ice, water
Conductive vests under gear at the start of shift
**Plan F (no access to power and no PPE removal)**
Conductive vests under gear at the start of shift

Abbreviations: PPE, personal protective equipment.

The body cooling strategies can be divided into three categories: (a) pre‐cooling, (b) per‐cooling (during work), and (c) post‐cooling. Pre‐cooling would consist of implementing cooling strategies prior to the start of the work shift. As employers cannot dictate what occurs before or after work, employers are encouraged to educate workers on the positive effects of body cooling timing. For example, employers can inform workers that pre‐cooling strategies can be used to increase heat storage capacity before experiencing heat stress (i.e., their bodies are able to store more heat before experiencing the negative influence of heat) (Jones et al., 2012; Watkins et al., 2018). “Per‐cooling” is a term that refers to utilizing body cooling strategies during work or during rest breaks (Bongers et al., 2017). The objective of per‐cooling aims to attenuate the rise in core temperature and/or physiological strain during work (Bongers et al., 2017). Reductions in thermal strain have been shown to improve exercise performance in athletes (Bongers et al., 2017) Similar improvements in performance (i.e., productivity) may occur in workers who are exposed heat stress. Lastly, post‐cooling enhances the physiological recovery process following a work shift in the heat (Brearley & Walker, 2015). These strategies can be implemented immediately following the work shift or at home if the workers choose to. Current research examining the timing of cooling strategies in occupational settings tends to be limited to per‐cooling strategies (Chicas et al., 2020; McEntire et al., 2013).

##### 4.6.2.1 Considerations

Employers must educate themselves and their employees on the effectiveness of body cooling methods and the consequences that may stem from use. For example, if a body cooling device improves the workers' perceived thermal comfort, but fails to adequately reduce their core temperature, workers may overestimate their ability and begin to “work harder” as no changes in heat storage have been made in their bodies (Vargas et al., 2019). This false perception of the success of cooling would further increase their risk of heat‐related illness (Casa et al., 2015). Other considerations include body cooling for as long as possible to achieve optimal benefits. For example, employers should encourage workers to utilize body cooling strategies for as long as possible to receive its benefits (i.e., reduce heat‐related illness risk). Lastly, employers must consider factors that affect the efficacy of various cooling methods. For example, the inserts for ice vests must be replaced or hand cooling modalities must be recharged to achieve optimal benefit. Therefore, they will need to determine whether these cooling modalities can be appropriately implemented at their worksite.

#### 4.6.3 Gaps in Knowledge

1. Acceptability and feasibility of implementation of cooling strategies within each sector to identify corresponding barriers and facilitators.
2. Interactions between different cooling interventions and other preventative strategies (i.e., personal protective clothing and gear, administrative controls, HA).
3. Cooling interventions must be tailored to serve the overall population of workers (i.e., consider age, sex, culture, disease, geographical location, activity level) within their work‐specific setting.

### 4.7 Textiles and Personal Protective Gear

#### 4.7.1 Background and Significance

PPE serve as defense against multiple occupational hazards (physical, chemical, and electrical) in various settings (firefighting, police, military, chem/bio, mining, welding, agriculture, construction, etc.). However, the clothing that serves to protect from other occupational hazards can also exacerbate heat‐related illness and increases risk of injury and fatality as PPE restricts the flow of heat and vapor from the body to the external environment (McLellan & Havenith, 2016). The added weight, bulk, and insulation of PPE increase the onset of heat strain, especially when strenuous work is performed in hot and humid conditions (Havenith, 1999; Watson et al., 2019; Yeargin et al., 2006). Material structure, garment design, and garment fit play key roles in the buildup of metabolic heat (Jin et al., 2018).

#### 4.7.2 Current Research

##### 4.7.2.1 Material Considerations

The materials incorporated in personal protective clothing vary widely from extremely lightweight disposable nonwovens to thick, heavy, dense woven fabrics necessary for protecting the wearer from heat, flame, projectiles, and sharp objects. All pose risks from an occupational heat stress standpoint and these materials are often treated or combined with impermeable or semi‐permeable films to provide liquid and chemical protection. Such finishes and films, especially when worn in combination with other layers, block both convective and evaporative heat transfer from the body to the external environment, increasing risk of heat‐related illness. On the material level, ways to improve physiological comfort have been explored including incorporating phase change materials (PCMs) (Butts et al., 2017; McFarlin et al., 2016), wicking and moisture management treatments, and infrared heat reflective finishes. The majority of these textile finishes, however, have proven to be ineffective for occupational applications due to the excessive amounts of treatment needed, which negates the benefits due to added material weight, as well as the relatively short period of time for which they are effective.

Further, while fiber type is of importance for certain moisture management properties that may help the skin to feel cooler, the volume of air trapped within the fabric is much greater than the volume of fibers. Hence, clothing insulation (*I*_t_) is far more dependent on fabric thickness than fiber content. This insulation is essential for protection but interferes with heat loss and leads to heat stress. Methods for reducing heat stress through materials should be considered by providing alternative insulation mechanisms, such as shape memory alloys which expand when needed for thermal protection and allow for reduced clothing layers, thereby increasing heat transfer (He et al., 2018; Jin et al., 2018). Lighter weight fibers, novel fabric materials, and multi‐layer composites may also lead to reduced ensemble weight and metabolic burden, resulting in a slower onset of heat strain (McQuerry et al., 2020). Improving movement efficiency through the adoption of novel stretch materials (i.e., flame‐resistant knits and one‐ or two‐way stretch membranes) may also reduce the wearer's thermal burden (McLellan & Havenith, 2016; McQuerry et al., 2018a, 2018b).

##### 4.7.2.2 Design Considerations

In humans, heat and vapor transfer are hindered by the clothing layers, the air enclosed within those layers, and the still air bound to the outermost layer (Havenith, 1999). Design modifications for enhancing heat loss through these layers include ventilation (both passive and active) (Bouskill, 1999; Lumley et al., 1991; McQuerry et al., 2018a, 2018b; Reischl & Stransky, 1980), active cooling devices (Bach et al., 2019; Chicas et al., 2020; Tokizawa et al., 2020) and systems modularity which involves deploying certain layers of the ensemble for specific activities (McQuerry et al., 2020). For example, for firefighting protecting clothing, a single‐layer garment may be worn for search and rescue activities in lieu of the multi‐layer system (Jin et al., 2018). Body sweat mapping should also be used for optimum placement of design features (i.e., vents, stretch materials, PCMs, etc.) and reinforcements (reflective trim, pockets, labels, etc.) to enhance evaporative heat loss and increase mobility (Watson et al., 2019).

The impact of fit and the amount of ease built into the garment should not be ignored as anthropometric factors influence insulation and subsequent heat transfer (McLellan & Havenith, 2016; Wang et al., 2012). In general, tighter‐fitting clothing provides less heat transfer resistance than loose‐fit clothing with Havenith et al. (1990) observing that work clothing had a 6%–31% lower insulation when designed to fit closer to the body. Moreover, garments provided by employers are often designed for men only and do not account for anthropometrics differences between sexes. Females may have clothing that is loose fitting and therefore, greater insulative properties (Havenith et al., 1990; Park & Langseth‐Schmidt, 2016).

##### 4.7.2.3 Testing Considerations

The PPE selection process should involve testing of both materials and full systems ensembles to ensure a realistic understanding of the resistance to heat loss when clothing is worn by the user. Standards such as those by the National Fire Protection Association (NFPA) require total heat loss (THL) testing for gear to be certified and distributed (NFPA 1584, 2015). This testing, however, is heavily limited by its lack of a full systems ensemble approach. For example, THL and thermal protective performance are only assessed on the material level and do not consider garment reinforcements, air gaps, and fit which come into play when a two‐dimensional fabric is sewn into a three‐dimensional garment and worn on the human body (McQuerry, DenHartog, & Barker, 2018) [163]. Instead, when possible, data should be collected on the full systems ensemble through the use of instrumented sweating thermal manikins and human wear studies to capture more realistic heat loss and heat strain data on the three‐dimensional form (Psikuta et al., 2017).

There are no standards for the proper use of many types of occupational PPE to allow for adequate recovery and prevent heat stress. Even when such standards do exist, like NFPA 1584 (Kim et al., 2019; NFPA 1584, 2015), they are not always followed. Removing PPE during recovery periods is the simplest and easiest cooling method (Kim et al., 2019) especially as core temperature continues to rise after the completion of activity (Horn et al., 2011).

Another important PPE consideration is the garments worn during physical fitness testing for employment. A common issue with physical employment tests is that they simulate the weight of PPE as opposed to requiring the actual PPE for the job to be worn. The addition of weight alone is not a substitute for the multi‐layered garment and accessories that are required to do the job (Havenith, 1999). Such testing underestimates the metabolic demand of PPE as it does not consider the reductions in movement efficiency or the increased resistance in thermal and evaporative heat loss when PPE is worn (Havenith, 1999). Moreover, wearing PPE ensembles during the later stages of HA is necessary to accurately estimate the thermal strain of work.

#### 4.7.3 Gaps in Knowledge

1. The assessment of low‐level risk PPE (e.g., mining, agricultural, construction, etc.) for occupations that are less regulated compared to first responder and military applications (e.g., migrant agricultural workers brought to the United States on H2A visas must be provided food and shelter, yet there are no requirements for providing clothing to protect from heat stress, pesticide application, and long‐term UV exposure).
2. The impact and performance of male‐designed gear when used by female workers.
3. The development of material and garment performance guidelines to help end users select the most appropriate PPE for heat stress reduction.

### 4.8 Emergency Procedures and EAPs

#### 4.8.1 Background and Significance

Medical EAPs contain vital information on how to initiate responses during a potentially catastrophic event. To complement EAPs, workplace manuals should contain policies and procedures that address heat‐related illnesses. While best practices regarding the treatment of a heat‐related injury may be fully established in the medical literature (Belval et al., 2018; Casa et al., 2012, 2015; Demartini et al., 2015), it is clear that mandated policies and EAP policies facilitate and improve step‐by‐step execution of these standards during an emergency (Drezner et al., 2013; Kerr et al., 2019; Scarneo‐Miller et al., 2020). Education of, access to, communication of, and rehearsal of these procedures is necessary and should involve all stakeholders (Andersen et al., 2002; El‐Shafei et al., 2018; Price et al., 2018). While heat‐related emergencies are rarely predictable, when they do occur, the response that occurs in the first 5–10 min will likely dictate outcome (Belval et al., 2018; Casa et al., 2012; Courson, 2007; Drezner et al., 2013). Employers have a professional responsibility to create a medical EAP and may have a legal duty. The Occupational Safety and Health Act of 1970 mandates that all nongovernment employers provide a safe and healthful workplace for their workers (*OSH act of 1970*, OSHA, 1970). Employers have to evaluate if a particular job or situation exposes a worker to recognized hazards and remove or protect workers from those hazards (OSHA, 1926.23, n.d. OSH act of 1970, OSHA, 1970). In event of an emergency, the employer must provide the employee with access to prompt and appropriate care. For EHS victims, a thorough EAP policy and procedures section on heat‐related illnesses may facilitate rapid recognition and assessment, leading to early intervention with rapid, aggressive cold water immersion, improving the outcome and recovery from EHS (Adams et al., 2016; Demartini et al., 2015; McDermott et al., 2007; Stearns et al., 2016).

##### 4.8.1.1 EAP Development and Policy and Procedures Content

Current OSHA standards addressing exit routes and emergency planning dictate that an EAP must be in place (Title 29, Code of Federal Regulations Part 1910, Subpart E—Exit Routes and Emergency Planning, 1910.38), but OSHA does not detail requirements for employers to create EAPs that address heat‐related emergencies. Nor do current OSHA standards address workplace heat hazards. However, NIOSH guidance suggests within the heat stress safety data sheet that emergency and first aid procedures, including site‐specific contact information be included (NIOSH, 2016). OSHA guidance on heat index usage provides the most in depth description of what should be outlined to prepare for an occupational heat emergency (Table 8).

**Table 8:** OSHA Preparation Recommendations to Employers for Heat‐Related Illness

**Recommendation**
Create first aid and emergency action plan for heat‐related illness
Train supervisors and workers on the signs and symptoms of heat‐related illness and emergency response procedures
Be prepared to provide first aid for any heat‐related illness and call emergency services (e.g., call 911) if a worker shows signs and symptoms of heat stroke
Be able to provide clear and precise directions to the worksite
Immediately respond to symptoms of possible heat‐related illness—move the worker into the shade, loosen the clothing, wet and fan the skin, place ice‐packs in the armpits and on the neck. Give the worker something to drink. Call emergency services if the worker loses consciousness or appears confused or uncoordinated. Have someone stay with an ill worker
Alert employees and supervisors of high heat periods
Develop a plan to reschedule or terminate work if conditions become too risky

Source: OSHA. Using the heat index: A guide for employers [6].

Abbreviations: OSHA, Occupational Safety and Health Administration.

While the OSHA recommendations in Table 8 provide a general basis for the need to have an EAP, it does not call for or describe the items that should be included in the EAP. It should be highlighted that there is no standard that requires the employer to have a written policy and procedures section in their workplace manuals for managing serious and/or potentially life‐threatening work‐related injuries, but we believe that it is imperative for worker safety and falls under OSHA standard 1926.23 “*First aid and medical attention*” and 1910.151 “*Medical services and first aid*” (OSHA, 1910.151, 1998; OSHA, 1926.23, n.d.). While emergencies and accidents are not predictable, based on extensive use and development of EAPs in the sport sector, the authors suggest the following key areas and content be included in a medically centered EAP for application within the occupational setting (Table 9).

**Table 9:** Components and Standards of a Medical Emergency Action Plan (EAP) for Occupational Settings

1	The EAP is developed and coordinated with local EMS, company safety officials, and any onsite medical personnel
2	The EAP is distributed in appropriate languages and reviewed by all workers annually in addition to upon the start of employment
3	Each location (lab, active work site, etc.) that employees work has its own location specific EAP
4	The EAP identifies location of onsite emergency equipment
5	The EAP identifies personnel and their responsibilities to carry out the plan of action with designated chain of command
6	The EAP lists contact information for EMS and other key personnel, as well as facility address, location, GPS coordinates
7	The EAP provides recommendations for documentation that should be taken after a catastrophic incident
8	The EAP is rehearsed annually by employees and other pertinent medical personnel. In workplaces with high turnover, the EAP should be rehearsed more often
9	The EAP includes information for health care professionals providing medical care which is included in the review and rehearsal
10	The EAP is updated annually by all relevant employees
11	The EAP is posted at every worksite in languages understood by employees

Once an EAP is established, it is imperative that it is implemented effectively via language‐appropriate posted copies/distribution, education on the EAP for all personnel, and routine rehearsals of the EAP. Please see Appendix D in supporting information for EAP and EHS specific policy and procedures template that can be customized for worksites.

#### 4.8.2 Current Research

To our knowledge, there are no investigations examining the effectiveness of EAPs on EHS outcomes and recovery in the occupational setting. Literature surrounding the effectiveness of EAPs is most often found in school, athletic, and military settings (Courson, 2007; Drezner et al., 2009; Scarneo et al., 2019). For example, an epidemiological investigation in the US high schools reported that implementation of automated external defibrillator (AED) programs, which included an EAP for cardiac arrest, resulted in higher survival rates following sudden cardiac arrest compared to the US high schools that did not implement this program (Drezner et al., 2013). More research is warranted to quantify the effect of EAPs for EHS in the occupational setting.

##### 4.8.2.1 EHS and Heat‐Related Illness Recovery Protocols

Worksites and jobs that pose a risk for workers to succumb to a heat‐related illness should have a heat‐related illness prevention program that includes how to safely return those workers following their injury (McDermott et al., 2007). Heat‐related illnesses such as heat syncope and exertional heat exhaustion should be recorded and followed up with regardless of severity (McDermott et al., 2007). Recording these events is critical to assess risk factors for future prevention strategies. As EHS is a medical emergency, a standardized return to work protocol must be in place to evaluate whether the worker is healthy enough to return (McDermott et al., 2007). A minimum of 2 weeks of complete rest followed by a professional clinical assessment by a physician, to include lab work to verify end organ enzyme levels have returned to normal, is called for (McDermott et al., 2007). Once the workers' clinical status and laboratory values have returned to normal, the worker should perform rehabilitation ideally under the direction of a medical professional who is trained to monitor, identify, and treat EHS (McDermott et al., 2007). Employers and workers should note that return to work guidelines for heat‐related injuries and illnesses are not specific to work settings (i.e., specific to athletics). As previous catastrophic best practice mandates in athletics have shown, implementing best practices is significantly enhanced when a mandate is in place for those condition‐specific policies and the mandate includes presence of EAPs (Courson, 2007; Drezner et al., 2013; Kerr et al., 2019; Scarneo et al., 2019). As such, leading organizations must mandate the use of medically specific EAPs at worksites and create a plan to return workers following EHS with the guidance of medical professionals.

#### 4.8.3 Gaps in Knowledge

1. Current use of medically specific EAPs is unknown in the workforce or industrial sectors.
2. Standardized post‐EHS assessment, graduated return to activity and onsite medical monitoring, returning workers safely from an EHS event (i.e., currently remains open to employer decisions).

## 5 Conclusion

This document presents evidence‐based and feasible occupational heat safety recommendations that are intended to serve as a foundation for heat safety recommendations in the occupational setting. Safety managers, industrial hygienists, and employers can utilize these recommendations and tailor them to their respective workplace based on the occupational setting. A major strength of the document was the use of the Delphi method to develop each recommendation. This methodological approach produced occupational heat safety recommendations that were created and systematically scored (scientific evidence, feasibility, clarity) among 51 inter‐disciplinary experts. Twelve of the 51 experts were safety managers (24%) who were responsible for safety initiatives within their organization. Their involvement in the creation and scoring of each recommendation was critical to address the feasibility (i.e., likelihood of adoption) of each recommendation. Future updates to this document should include level of adoption as a scoring category and should include a larger sample of safety managers and workers across a variety of different occupations.

Although this document presents effective prevention strategies to mitigate heat strain and protect workers from heat‐related illnesses, it is important to recognize that despite implementing best‐practice and comprehensive heat safety plans, no safety plan is failproof. Employers and workers should have training on the signs and symptoms and emergency response procedures for EHS to prevent unnecessary deaths. Licensed medical professionals such as EMS are responsible for diagnosis and treatment of EHS at the workplace. If there are medical professionals at the workplace, guidelines related to recognition, diagnosis and treatment of EHS based on the occupational setting can be found in Table S3.

## Disclaimer

The findings and conclusions in this report are those of the authors and do not necessarily represent the official position of the National Institute for Occupational Safety and Health, Centers for Disease Control and Prevention. The views expressed in this abstract/manuscript are those of the authors and do not reflect the official policy or position of the Department of the Army, Department of Defense, or the US Government.

## Conflict of Interest

The authors declare no conflicts of interest relevant to this study.

## Supporting information

Supporting Information S1

## Data Availability

Delphi method scoring data are available at Margaret Morrissey. (2021). *Delphi method scoring for consensus statement for occupational heat safety* [Data set]. In GeoHealth (Version 1). Zenodo. https://doi.org/10.5281/zenodo.5075944
