# Supplementary material for: Heat Safety in the Workplace: Modified Delphi Consensus to Establish Strategies and Resources to Protect the US Workers
Source: Geohealth. 2021 Aug 1;5(8):e2021GH000443. doi: 10.1029/2021GH000443 (PMC8388206; doi:10.1029/2021GH000443)
Supplement: Supplementary file 1 — Supporting Information S1 [file GH2-5-e2021GH000443-s001.docx]

**Appendix A**

| **Table S1. Task Force Members for Occupational Heat Safety Round Table on December 10^th^, 2020** | |
| --- | --- |
| **Task Force Member** | **Affiliation** |
| William Adams, PhD, ATC | *University of North Carolina at Greensboro* |
| Sarah Anderson | *Magid* |
| Thomas Bernard, PhD | *University of South Florida* |
| Courteney Benjamin, PhD., CSCS | *Samford University* |
| Robin Birk | *United States Steel* |
| Matthew Block | *Magid* |
| Gabrielle Brewer, MA | *Korey Stringer Institute, University of Connecticut* |
| Bruce Cadarette | *United States Army Research Institute of Environmental Medicine* |
| Douglas Casa, PhD | *Korey Stringer Institute, University of Connecticut* |
| Brittany Cohen | *Magid* |
| Gary Cohen | *Magid* |
| Calvin Croftcheck | *United Steel International* |
| David DeGroot, PhD, FACSM | *Army Heat Center, Fort Benning GA* |
| Juley Fulcher, JD, PHD | *Public Citizen* |
| Andreas Flouris, PhD | *University of Thessaly* |
| Jennifer Garza, ScD | *University of Connecticut* |
| Andrew Grundstein, PhD | *University of Georgia* |
| Joshua Hamm | *First Line Technology* |
| Joshua Hockett | *Booz Allen Hamilton* |
| Yuri Hosokawa, PhD, ATC | *Waseda University* |
| David Hostler, PhD | *University atBuffalo* |
| Robert Huggins, PhD, ATC | *Korey Stringer Institute, University of Connecticut* |
| Brenda Jacklitsch, PhD, MS | *National Institute for Occupational Safety and Health* |
| John Jardine, MD | *Korey Stringer Institute* |
| Ollie Jay, PhD, FACSM | *University of Sydney* |
| Tony Kalogridis | *H & A Farms* |
| Stavros Kavouras, PhD, FACSM, FECSS | *Arizona State University* |
| John Kimball | *Tesla* |
| Kurt Krueger, M.S., MBA | *ORC-Health, Safety & Environmental (HSE)* |
| Elaine Lee, PhD | *University of Connecticut* |
| Rebecca Lopez, PhD, ATC, CSCS | *University of South Florida* |
| Ronda McCarthy, MD | *Concentra* |
| Brendon P. McDermott, PhD, ATC, FACSM | *University of Arkansas* |
| Meredith McQuerry, PhD | *Florida State University* |
| Margaret C. Morrissey, MS | *Korey Stringer Institute, University of Connecticut* |
| Nathaniel Nye, MD | *Joint Base San Antonio - Lackland* |
| Yannis Pitsiladis, PhD | *University of Brighton* |
| Riana Pryor, PhD, ATC | *University atBuffalo* |
| Brent Ruby, PhD, FACSM | *University of Montana* |
| Zachary Schlader, PhD | *Indiana University* |
| Marc Schlenker, MD, MPH | *University of California, Davis* |
| Caroline Smith, PhD, FACSM | *Appalachian State University* |
| Denise Smith, PhD | *Skidmore College* |
| June Spector, MD, MPH | *University of Washington* |
| Michael Szymanski, MS, ATC | *Korey Stringer Institute, University of Connecticut* |
| Chris Valletta | *MISSION* |
| Jennifer Vanos, PhD | *Arizona State University* |
| Nicole Vargas, PhD | *University of Sydney, Australia* |
| Jon Williams, PhD | *National Institute for Occupational Health and Safety* |
| Susan Yeargin, PhD, ATC | *University of South Carolina* |

Appendix B

| **Table S2. Strategies and Resources to Assist with Implementation** |
| --- |
| **HEAT HYGIENE** |
| *Physical Examinations (Rec #1):* if physical examinations are not mandated by the workplace, employers are encouraged to conduct a pre-placement and annual medical survey to screen for conditions associated with increased risk of heat-related illness (Table S2) |
|  |
| *Wellness Program:* An effective wellness program is one that incorporates an integrated and holistic approach such as Total Worker Health. An example of the Total Worker Health program can be found at <https://www.cdc.gov/niosh/twh/default.html>. |
|  |
| *Educational Materials:* List of resources to find training and educational materials: <https://www.osha.gov/heat>, <https://www.cdc.gov/niosh/topics/heatstress/default.html>, |
|  |
| *Onboarding (Hiring) Training:* Employers or supervisors should communicate (verbally, in a training video, etc) the concept of the “buddy system” and ensure that workers understand to look out for one another on the job site. |
|  |
| *Working Alone:* In situations when employees work alone (e.g. Postal carriers), instead the “buddy system” cannot be implemented, they should have access to a cell phone to alert their supervisor or emergency contact in time of need. |
|  |
| **HYDRATION** |
| *Hydration supplies to consider:* drinking coolers, disposal cups, electrolyte hydration mix or gel, electrolyte-carbohydrate beverages, individual water bottles, and wearable hydration packs. |
|  |
| *Urine Color Assessment:* a validated, urine color chart can be found at hydrationcheck.com. It is important to use a validated chart to accurately assess hydration based on urine color.   1. Armstrong L.E., Maresh C.M., Castellani J., Bergeron M., Kenefick, R. W., LaGasse, K.E., Riebe, D. Urinary indices of hydration status. International Journal of Sport Nutrition 4:265-279, 1994. 2. Armstrong L.E., Herrera Soto J.A., Hacker F.T., Casa D.J., Kavouras S.A., Maresh C.M. Urinary indices during dehydration, exercise, and rehydration. International Journal of Sport Nutrition 8:345-355, 1998. 3. Armstrong L.E. Assessing hydration status: The elusive gold standard. Journal of the American College of Nutrition 26(5):575S-584S, 2007. |
|  |
| *Assessing Change in Body Weight:* If workers have access to a body weight scale to calculate their sweat rate, they can use the following equation to estimate how much sweat they have lost during their work shift:  Sweat rate(L/hr) = pre activity bodyweight – post activity bodyweight) + fluid intake – urine volume ]/exercise time in hours ([www.ksi.uconn.edu](http://www.ksi.uconn.edu))  Sweat rate can tell your workers how many liters they lost in sweat during an hour of work. If a worker lost 2 liters in an hour, their sweat rate would be 2 L/hr.  [Brendon P. McDermott, Scott A. Anderson, Lawrence E. Armstrong, Douglas J. Casa, Samuel N. Cheuvront, Larry Cooper, W. Larry Kenney, Francis G. O’Connor, and William O. Roberts (2017) National Athletic Trainers’ Association Position Statement: Fluid Replacement for the Physically Active. Journal of Athletic Training: September 2017, Vol. 52, No. 9, pp. 877-895.](https://www.ncbi.nlm.nih.gov/pubmed/28985128) |
|  |
| 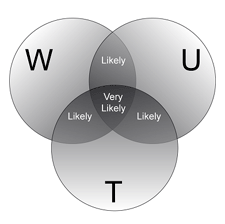*Hydration Assessment: WUT Venn Diagram Decision Tool:* Using the WUT Venn Diagram as a decision tool is a useful method that combines simple markers of hydration. This assessment tool combines the following three markers of hydration: weight (change in body weight), urine (urine color & urine frequency), and thirst. The Venn Diagram uses these markers to determine the likelihood that fluid intake is inadequate or not. If you are dehydrated in two of these assessments, fluid intake in likely inadequate, while dehydration in all three indicate that daily fluid intake is very likely inadequate.  Cheuvront, Samuel N., and Michael N. Sawka. "Hydration assessment of athletes." *Sports Sci Exchange* 18.2 (2005): 1-6. |
|  |
|  |
| **HEAT ACCLIMATIZATION** |
| *Does your worksite need a heat acclimatization plan?* Figure 1 provides a flow chart for safety managers to determine whether their worksite needs to implement a heat acclimatization plan. |
|  |
| *Duration and Intensity of Heat Acclimatization Program in Occupational Settings: Strategies* recommended by NIOSH can be found in Criteria for a Recommended Standard: Occupational Exposure to Heat and Hot Environments. |
|  |
| *Duration and Intensity of Heat Acclimatization Program in Athletic and Military:*  HA strategies or resources from military and athletic settings include:   1. Department of the Army Headquarters (2016) Prevention of heat and cold casualties. (Military) 2. Parsons, Iain T., Michael J. Stacey, and David R. Woods. "Heat adaptation in military personnel: mitigating risk, maximizing performance." *Frontiers in physiology* 10 (2019): 1485. (Military) 3. Pryor, J. Luke, et al. "Application of evidence-based recommendations for heat acclimation: individual and team sport perspectives." *Temperature* 6.1 (2019): 37-49. (Athletics) 4. Sekiguchi, Yasuki, et al. "Practical Implementation Strategies for Heat Acclimatization and Acclimation Programming to Optimize Performance." *Athletic Training and Sports Health Care* (2021). (Athletics) 5. Daanen, Hein AM, Sebastien Racinais, and Julien D. Périard. "Heat acclimation decay and re-induction: a systematic review and meta-analysis." *Sports Medicine* 48.2 (2018): 409-430. (Athletics) 6. Périard, J. D., Sebastien Racinais, and Michael N. Sawka. "Adaptations and mechanisms of human heat acclimation: applications for competitive athletes and sports." *Scandinavian journal of medicine & science in sports* 25 (2015): 20-38. (Athletics) |
|  |
| **ENVIRONMENTAL MONITORING** |
| *WBGT Cost:* WBGT devices range from approximately $100-$3,000 dollars depending on the size, accuracy, and portability. |
|  |
| *Examples of WBGT Devices:* Kestrel 4400 Heat Stress Tracker, QUESTemp°34, Extech HT30 Heat Stress Wet Bulb Globe Temperature Meter, General WBGT8778 Heat Index Checker, REED Heat Index WBGT Meter model SD-2010, and WBGT-103 Heat Stroke Checker.  Cooper, Earl, et al. "An evaluation of portable wet bulb globe temperature monitor accuracy." *Journal of athletic training* 52.12 (2017): 1161-1167. |
|  |
| *Activity Modifications Based on Environmental Conditions:* Activity modifications specific to occupational settings are presented by NIOSH and ACGIH  Hosokawa, Yuri, et al. "Activity modification in heat: critical assessment of guidelines across athletic, occupational, and military settings in the USA." *International journal of biometeorology* 63.3 (2019): 405-427. |
|  |
| **PHYSIOLOGICAL MONITORING** |
| *How do I know if a physiological sensor or device is validated?* Validity is the extent to which the scores from a measure represent the variable they are intended to. To measure the validity of an assessment for your workplace, its scores are compared to the “gold standard” measure in the same worker population, setting, AND at the same intensity. For example, an estimated core temperature app must be compared to esophageal or rectal thermometry. Employers are encouraged to review scientific literature or consult with scientists to determine valid physiological monitoring devices for their specific worksite. |
| *How do I know if a physiological sensor or device is reliable?* Reliability refers to the consistency of a measure. It must be reliable over time (test-retest reliability), across items (internal consistency), and across different users (inter-rater reliability, if appliable). Employers are encouraged to review scientific literature or consult with scientists to determine reliable physiological monitoring devices for their specific worksite. |
|  |
| **BODY COOLING** |
| *Important Safety Considerations of Cooling Effects:* Using body cooling strategies that have low efficacy result in the perception of feeling “cool” with no physiological benefits (I.e. reduction in core temperature). If the worker feels "cooler” without reducing heat strain, they may perform at a higher intensity (or for longer duration), which could further increase their risk of heat-related illness. Duration and intensity of cooling are critical to reduce risk of heat-related illness.   1. Casa, Douglas J., et al. "National Athletic Trainers' Association position statement: exertional heat illnesses." *Journal of athletic training* 50.9 (2015): 986-1000. 2. Armstrong, Lawrence E., et al. "Exertional heat illness during training and competition." *Medicine & Science in Sports & Exercise* 39.3 (2007): 556-572 |
|  |
| *Resources for choosing the correct cooling strategies for your worksite:*   1. Table 5 and 6, Figure 3 (Current paper) 2. Foster, Josh, et al. "Occupational heat stress and practical cooling solutions for healthcare and industry workers during the COVID-19 pandemic." Annals of work exposures and health 64.9 (2020): 915-922. (Occupational) 3. McEntire, Serina J., Joe Suyama, and David Hostler. "Mitigation and prevention of exertional heat stress in firefighters: a review of cooling strategies for structural firefighting and hazardous materials responders." Prehospital Emergency Care 17.2 (2013): 241-260. (Occupational) 4. Brearley, Matt. "Cooling methods to prevent heat-related illness in the workplace." Workplace health & safety 64.2 (2016): 80-80. (Occupational) 5. Chicas, Roxana, et al. "Cooling intervention studies among outdoor occupational groups: A review of the literature." American Journal of Industrial Medicine 63.11 (2020): 988-1007. (Occupational) 6. Adams, William M., Yuri Hosokawa, and Douglas J. Casa. "Body-cooling paradigm in sport: maximizing safety and performance during competition." Journal of sport rehabilitation 25.4 (2016): 382-394. (Athletics) |
|  |
| **TEXTILES AND PPE** |
| *Insulative Properties of Clothing for Environmental Monitoring:* UTCI and PET (environmental monitoring indices) utilize clothing adjustments to estimate thermal strain. Employers and supervisors should be aware of the insulative properties of the gear they provide their workers if they plan to use these indices.  Simple Calculation for Clothing and Thermal Insulation: https://www.engineeringtoolbox.com/clo-clothing-thermal-insulation-d_732.html |
|  |
| *Fit-testing and Certification for PPE:* OSHA guidelines state that the relative workplace exposure level determines what constitutes an acceptable fit for PPE and which fit test procedure is required. PPE certification is dependent on the occupation and is associated with governing bodies and regulatory agencies such as NIOSH, NFPA, AAMI, and ANSI. |
|  |
| **EMERGENCY ACTION PLANS** |
| *Medical Conditions That Can be Caused by Heat:* exertional heat stroke, cardiac events, liver injury, and kidney injury (examples) |
|  |
| *Sample Emergency Action Plan:* The following medical conditions related to heat require medical clearance prior to returning to work: A sample of an EAP for the worksite can be found in Appendix D. |
|  |
| *CPR (Cardiopulmonary Resuscitation), First Aid, and AED (Automated External Defibrillator) Certification:* <https://www.redcross.org> provides classes that train 9 million people a year in life-saving skills. Supervisors and workers are encouraged (if they are not required) to be CPR, First Aid, and AED certified to reduce the risk of fatalities associated with heat and/or related to work. |
|  |
| ***OTHER HELPFUL RESOURCES*** |
| 1. Morris, Nathan B., et al. "The HEAT-SHIELD project—Perspectives from an inter-sectoral approach to occupational heat stress." *Journal of Science and Medicine in Sport* (2021) 2. Flouris, Andreas D., et al. "Workers' health and productivity under occupational heat strain: a systematic review and meta-analysis." *The Lancet Planetary Health* 2.12 (2018): e521-e531. 3. Casanueva, Ana, et al. "Escalating environmental summer heat exposure—a future threat for the European workforce." *Regional Environmental Change* 20.2 (2020): 1-14. 4. Nybo, Lars, et al. "Global heating: Attention is not enough; we need acute and appropriate actions." (2017): 199-201. 5. Notley, Sean R., Andreas D. Flouris, and Glen P. Kenny. "Occupational heat stress management: Does one size fit all?." *American journal of industrial medicine* 62.12 (2019): 1017-1023. 6. Tustin, Aaron W., et al. "Evaluation of occupational exposure limits for heat stress in outdoor workers—United States, 2011–2016." *Morbidity and Mortality Weekly Report* 67.26 (2018): 733. 7. Tustin, Aaron W., et al. "Risk factors for heat-related illness in US workers: an OSHA case series." *Journal of occupational and environmental medicine* 60.8 (2018): e383-e389 8. Morris, Nathan B., et al. "Health vs. wealth: Employer, employee and policy-maker perspectives on occupational heat stress across multiple European industries." *Temperature* (2020): 1-18. |
|  |

**Appendix C**

| **Table S3. Recognition and Treatment of Exertional Heat Illnesses and Emergency Medical Supplies Required Based on Work Setting** | | | | | |
| --- | --- | --- | --- | --- | --- |
| **Tier I:** Indoor setting or outdoor setting with close access to a facility that has power, storage capabilities for materials, etc. | | | | | |
| **Tier II:** Remote setting with a temporary facility with large group of workers (e.g., a trailer) | | | | | |
| **Tier III:** Remote Settings with Small Group of Workers (e.g., setting with no facility) | | | | | |
| **EHI** | **Recognition** | **Treatment Standard** | **Tier I Equipment** | **Tier II Equipment** | **Tier III Equipment** |
| **Heat Syncope (fainting)** | Brief episode of fainting, must rule out cardiac event; decreased pulse rate; collapse due to prolonged standing in the heat | - Remove the individual from the heat source, move to a shaded area - Elevate lower extremities - Remove excess clothing - Assess heart rate, breath rate, blood pressure - Superficial cooling | Water  Ice  Cooler  WBGT Device  Fan  Fan with mist  Towels for cooling  Electrolyte drink*  Pulse oximeter  Smartwatch* | Water  Ice  Cooler  Battery-operated fan  WBGT Device  Tarp (to use for shade if no shade available)  Pulse oximeter  Smartwatch* | Water  Ice  Cooler  Light Tarp/pop up tent (to use for shade if no shade available)  WBGT Device  Ice Towels  Pulse oximeter  Smartwatch* |
| **Exertional Heat Exhaustion** | Inability to complete tasks, fainting, extreme fatigue, headache, dizziness, low blood pressure, vomiting, | - Obtain rectal temperature to rule out EHS (<40.5°C/105°F) - Remove individual from the heat source, move to a shaded area - Remove excess clothing, expose as much skin to promote evaporative cooling - Superficial cooling (Provide ice towels, fanning) - Assess heart rate, breath rate, blood pressure | Rectal Thermometer  Pulse oximeter  Portable tub/Tarp  Water  Ice  Cooler  Fan  Fan with mist  Ice towels  Electrolyte drink  Smartwatch* | Rectal Thermometer  Pulse oximeter  Portable tub/Tarp  Water  Ice  Cooler  Fan  Fan with mist  Ice towels  Electrolyte drink*  Smartwatch* | Rectal Thermometer  Water  Ice  Cooler  Light Tarp/pop up tent (to use for shade if no shade available)  Ice Towels  Electrolyte drink  Pulse oximeter  Smartwatch* |
| **Exertional Heat Stroke (EHS)** | Severely impaired cognitive function  Core body temperature >40.5C/105F,  Disorientation, bizarre behavior, emotional responses, weakness, nausea, fainting, vomiting, dizziness, light-headedness, irrational behavior, headache, hyperventilation, diarrhea, collapse, staggered movement, and altered consciousness | - Obtain rectal temperature to confirm EHS (>40.5° C/105°F) - • Activate Emergency Action Plan/Advanced Medical Care - Lower body temperature as quickly as possible, within 30 minutes of recognition - Whole-body Cold-water Immersion - Water temperature should be 35-59°F - Continuous stirring/agitation of water - **Do not remove person from active cooling until core temperature reaches 102.5**°**F** | Rectal Thermometer  WBGT Device  Pulse oximeter  Portable tub/Tarp  Water  Ice  Cooler | Rectal Thermometer  WBGT Device  Pulse oximeter  Portable tub/Tarp  Water  Ice  Cooler | Rectal Thermometer  WBGT Device  Pulse oximeter  Portable tub/Tarp  Water  Ice  Cooler |
| **Exertional Hyponatremia** | Weight gain, Severely impaired cognitive function, Core body temperature < 40.5C/105F, disorientation, intense headache, vomiting, nausea, bizarre behavior, emotional responses, possible puffy fingers and toes | - Remove individual from heat source - • Activate Emergency Action Plan/Advanced Medical Care - Obtain rectal temperature to rule out EHS (<40.5/105F) - Serum sodium levels <129-135 mEq/L** - Asymptomatic and mild symptomatic hyponatremia symptoms should be treated with ***fluid RESTRICTION*** and salty foods - Severe cases require immediate activation of advanced medical care - Assess heart rate, breath rate, blood pressure | Rectal Thermometer  Salty foods (pickles, mustard, chips, pretzels, bouillon cubes)  Pulse oximeter  Smartwatch* | Rectal Thermometer  Salty foods (pickles, mustard, chips, pretzels, bouillon cubes)  Light Tarp/pop up tent (to use for shade if no shade available)  Pulse oximeter  Smartwatch* | Rectal Thermometer  Salty foods (pickles, mustard, chips, pretzels, bouillon cubes)  Light Tarp/pop up tent (to use for shade if no shade available)  Pulse oximeter  Smartwatch* |

Note: *Not required but useful; **If a serum electrolyte analyzer device is available (eg., ISAT)

**Appendix D**

Hello!

The following document is a template for the creation of an exertional heat stroke emergency action plan for your organization and has been developed based off of the [2002 “National Athletic Trainers’ Position Statement: Emergency Planning in Athletics](https://www.nata.org/sites/default/files/EmergencyPlanningInAthletics.pdf)”. This document is provided to create a personalized and comprehensive emergency action plan (EAP) for your organization.

Please note:

1. This document is simply a template to help you get started to create an EAP. Please revise, remove, add items as you wish.
2. The “EAP Template Fill-in” will provide you with the name of the section the information should be placed and the page numbers prior to entry of information. Please note that the page numbers can and will change as you start to put in your information into this template.
3. *Any words that are italicized and highlighted yellow are directions only and should be deleted prior to implementation of your EAP.*
4. Words within brackets such as [Organization Name] are highlighted yellow and should be revised to match your organization prior to implementation of your EAP.
5. The table of contents in this template is set using functions in Microsoft Word to match your document.
   1. When you have completed entering in the information, click on the table in the Table of Contents page, and click “Update Table”. Then click “Update Entire Table”; your table of contents should automatically be corrected.
   2. Should you choose to add sections to this document to match your organization’s needs, title the section as you wish, and then from the “Home” button on Microsoft Word, click “Heading 1”. This will then allow the table to recognize a new section when you update the table with the instructions from “a” above.

We hope you find this Emergency Action Plan template to be helpful. Please contact the Korey Stringer Institute with any questions you may have.

KSI Staff

*DISCLAIMER: This emergency action plan is not considered medical or legal advice. All content is for informational purposes, and the Korey Stringer Institute makes no claim as to accuracy, legality or suitability. They Korey Stringer Institute shall not be held liable for any errors, omissions or for damages of any kind.*

Exertional Heat Stroke

Emergency Action Plan

For [Name of Organization]

In case of an emergency, personnel responsibilities, locations of emergency equipment, and other emergency information such as 911 call instructions, addresses/directions to the site, and a chain of command with important phone numbers have been listed here.

Table of Contents

Table of Contents

[Personnel Involved in Development 4](#_Toc56425666)

[Documentation of Recent Changes 5](#_Toc56425667)

[Introduction 6](#_Toc56425668)

[Staff Education 10](#_Toc56425669)

[Chain of Command 10](#_Toc56425670)

[Emergency Telephone Numbers 11](#_Toc56425671)

[Emergency Situation Contact Tree 12](#_Toc56425672)

[Emergency Equipment Locations 13](#_Toc56425673)

[[ORGANIZATION NAME] Cold Water Tub Location 14](#_Toc56425674)

[General Plan of Action 15](#_Toc56425675)

[Emergency Action Procedures 16](#_Toc56425676)

[[Site Location] 16](#_Toc56425677)

[NAME OF VENUE 17](#_Toc56425678)

[Rehearsal Strategy 18](#_Toc56425679)

[Documentation of Seasonal Staff Educational Meeting 19](#_Toc56425680)

[Documentation of Emergency Equipment Maintenance 20](#_Toc56425681)

[Approval and Verification Page: 21](#_Toc56425682)

# Personnel Involved in Development

The following individuals were involved with the creation of this Emergency Action Plan:

*[delete for your own EAP the below is just an example]*

*Nancy Adams, MS, ATC (athletic trainer)*

*Brian Smith (site manager)*

*Michelle Jones, MA, RN (nurse)*

*Johnson City EMS*

# Documentation of Recent Changes

As changes to the EAP are made, please list the change, page affected and date that the change was made.

| **Specific Changes Made** | **Page(s) Affected** | **Date** |
| --- | --- | --- |
|  |  |  |
|  |  |  |
|  |  |  |
|  |  |  |
| **add rows as necessary* | | |

**EXERTIONAL HEAT STROKE EMERGENCY ACTION PLAN**

**OVERVIEW**

# Introduction

Exertional heat stroke (EHS) may occur during periods of intense physical work or hot conditions. Expedient action must be taken in order to provide the best possible care to the worker. The development and implementation of an emergency action plan will help ensure that the best care will be provided.

As emergencies may occur at any time and during any activity, all personnel must be prepared. All organizations have a duty to develop an emergency action plan that may be implemented immediately when necessary and provide appropriate standards of emergency care to all workers. This preparation involves formulation of an emergency action plan, proper medical access, maintenance of appropriate emergency equipment and supplies, utilization of appropriate emergency medical personnel, and regular updates to the EAP based on any updates to EHS care or workplace resources. While this may help to avert some potential emergencies, accidents and injuries may still occur, and proper preparation on the part of all personnel should enable each emergency situation to be managed appropriately and efficiently.

Components of an Emergency Action Plan

1. Emergency Personnel
2. Emergency Communication
3. Emergency Equipment
4. Roles of First Responder
5. Site Directions with a Map
6. Emergency Action Plan Checklist for Non-Medical Emergencies

**Emergency Personnel**

The first responder in an emergency situation is typically an onsite medical professional or EMS. However, the first responder may also be a colleague or another member of the organization.

Certification in cardiopulmonary resuscitation (CPR), first aid, automated external defibrillator (AED), emergency action plan review, and emergency plan review is required for all personnel. Copies of training certificates and/or cards should be maintained with your direct supervisor. *[revise above statement as determined by your organization/state]*

The emergency team may consist of physicians, emergency medical technicians, certified athletic trainers, managers, colleagues and possibly even bystanders. Roles of these individuals will vary depending on different factors such as site, personnel present, etc.

The four basic roles within the emergency team are:

1. **Establish scene safety and immediate care of the victim:**
   1. This should be provided by the most qualified individual present (the first individual in the chain of command).
2. **Activation of Emergency Medical Services:**
   1. This may be necessary in situations where emergency transportation is not already present at the site. Time is the most critical factor and this may be done by anyone on site.
3. **Equipment Retrieval:**
   1. May be done by anyone who is familiar with the types and locations of the specific equipment needed.
4. **Direction of EMS to the scene:**
   1. A designated individual should be in charge of meeting the emergency personnel as they arrive at the site. This person should have keys to all locked gates/doors.

**Activating Emergency Medical Services**

Call 9-1-1

Provide information

- Name, address, telephone number of caller
- Nature of the emergency (medical or non-medical)*
- Number of victims
- Condition of victim(s)
- First aid treatment initiated by first responder
- Specific directions as needed to locate the emergency scene (i.e. “use the south entrance off Asylum St.”)
- Other information requested by the dispatcher
- DO NOT HANG UP FIRST

*if non-medical, refer to the specified checklist of the organization’s emergency action plan

**Emergency Communication**

Communication is key to a quick, efficient emergency response. There is a pre-established phone tree to ensure all relevant parties are notified. Access to a working telephone line or other device, either fixed or mobile, should be assured. There should also be back-up communication in effect in case there is a failure of the primary communication. At every site it is important to know the location of a workable telephone. Please see page [fill in page # here] for emergency communication guidelines for [Organization Name].

**Medical Emergency Transportation**

For EHS cases, patients should be cooled on site (cool first, transport second) before transport. Any other emergency situation where there is loss of consciousness (LOC), or impairment of airway, breathing, or circulation (ABCs) or there is a neurovascular compromise should be considered a “load and go” situation and emphasis is placed on rapid evaluation, treatment, and proper transportation. Any emergency personnel who experiences doubt in their mind regarding the severity of the situation should consider a “load and go” situation and transport the individual.

**Post EAP Activation Procedures:**

*Documentation*

Documentation must be done by [Representative Name] immediately following activation of the EAP in accordance with OSHA and state standards.

*Debriefing*

The staff and leadership must evaluate the effectiveness of the EAP and conduct a staff debriefing. A specific timeline for changes to EAP should be made for promptness.

**Conclusion**

The importance of being properly prepared when emergencies arise cannot be stressed enough. Survival may hinge on the training and preparation of staff and healthcare providers. It is prudent to invest worker “ownership” in the emergency action plan by involving the staff. The emergency action plan should **be reviewed at least once a year** with all personnel and local emergency response teams. Through development and implementation of the emergency plan [Organization Name] helps ensure that the victim will have the best care provided when an emergency situation does arise.

# Staff Education

1. Each year, every manager will receive a copy of the Emergency Action Plan (EAP)
   1. Each worker will provide their signature to confirm they have read the documents and were able to ask any potential questions
2. A copy of the relevant EAP will be in each first aid medical kit which is to be kept at every site.
3. A copy of the EAP will be posted at every site.

# Chain of Command

The individual with the most medical training should always act as primary care-givers at the site of the injury or accident (when on-site) and would manage the situation according to the following rank:

1. *[place information from page 1 of EAP Fill-in document for chain of command with medical professionals present here]*

In the event that a medical professional not on-site at the time of injury, the following chain of command would be used:

1. *[place information from page 1 of EAP Fill-in document for chain of command without a medical professional present here]*

[Organization Name] CHAIN OF COMMAND

Most medically qualified


**this chain of command would be completed per team in the event that the chain is different per team*

# Emergency Telephone Numbers

This list is only to be used in case of an emergency.

| Off Worksite Contacts | Phone Number |
| --- | --- |
| Emergency |  |
| Police department |  |
| Fire and Ambulance |  |
| [Nearest] Hospital |  |
| [Second Nearest] Hospital |  |
| Hazardous Materials |  |
| Poison Control Center |  |

| Worksite Offices | Phone Number |
| --- | --- |
| Medical Services |  |
| Manager |  |
| Human Resources |  |
|  |  |
|  |  |

| Title | Name | Office | Cell |
| --- | --- | --- | --- |
| Athletic Trainer or Medical Personnel |  |  |  |
| EMS |  |  |  |
| Manager |  |  |  |
| Assistant Manager |  |  |  |
|  |  |  |  |

# Emergency Situation Contact Tree

First Responder on Scene

CARE

Life Threatening Condition

[1a] CALL

911

*Have [1b] get the AED

Call Spouse

Call Onsite Medical Professional

Call Manager

(if warranted)

Call

Onsite Medical Care

Provide care until EMS arrives.

Do not leave victim.

Provide **proper first aid** and **care** until EMS arrives.

Do not leave victim.

CARE

CARE

CARE

Non-Life Threatening Condition

CHECK

Call Supervisor

(if warranted)

After the situation is controlled, contact:

*[Site manager or supervisor Phone and Email]*

# Emergency Equipment Locations

***Emergency Equipment:***

*[List equipment here]*

- First Aid Kit, Emergency Bag, Biohazard Kit on site for events covered by medical professional
- First Aid Kit located with staff/manager
  - - 1. AED
         1. Located at every site
         2. Additional AED located outside [Organization Name] main entrance
      2. Nearest phone
         1. In [Organization Name] – there is a phone connected in the main office
      3. Cold Water Immersion Tub and water access
         1. In [Site Location]
         2. Water access via hose located at north end of site
      4. Ice
         1. In [Site/room Location]
      5. Rectal Thermometer
         1. In [Medical professional kit or supplies if applicable]
      6. Splints
         1. Located with each first aid kit
      7. Spine boards/Cervical Collar
         1. Will be provided by EMS upon arrival
      8. Bio-hazard Materials
         1. Red bags – in each first aid kit
         2. Disposal Bin – In room 123

# [ORGANIZATION NAME] Cold Water Tub Location

The main cold water immersion tub for [ORGANIZATION NAME] is located [place where the cold water tub is located]

*[Place the picture of your organization’s layout with the tub locations from page 5 of the “EAP Template Fill-in.” Below is an example of an organization layout with the AED placement. Delete this statement and picture]*


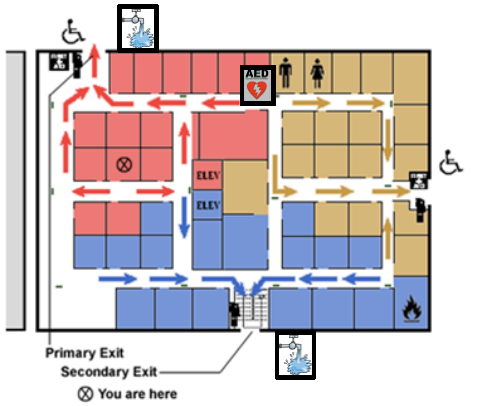


# General Plan of Action

1. Most medically qualified person will lead
2. Check the scene – is it safe to help?
3. Is the victim breathing? Conscious? Pulse?
   1. If NO, instruct person to call 911 – LOOK PERSON DIRECTLY IN EYES and

make sure they call!

Call contact safety officer if applicable

- 1. Check card for 911 call instructions for your location
  2. Perform emergency CPR/First Aid
  3. Instruct staff member to get AED

1. If severe bleeding – instruct individual to assist with bleeding control
2. If exertional heat stroke is suspected follow EHS EAP plan procedures
3. Instruct staff member to control crowd
4. Contact the medical professional of [ORGANIZATION NAME] if they are onsite but not at the scene
5. Contact spouse/significant other
6. Contact Manager and Supervisor
7. Instruct designated individual to meet ambulance to direct to appropriate site
8. Assist with care as necessary and continue until ambulance arrives
9. Document the event

**[ORGANIZATION NAME]**

# Emergency Action Procedures – Exertional Heat Stroke

## [Site Location]

*[Copy and paste this page multiple times for each site, you will have to change the address, directions and GPS coordinates for each site – delete this statement prior to use]*

**Activate the EAP if:**

- Any loss of consciousness
- Possible Exertional Heat Stroke
- Possible Spine Injury
- Dislocation, Open Fracture, Displaced Closed Fracture
- Difficulty or absent breathing or pulse
- Uncertainty of if you have a medical emergency

**Emergency Personnel:**

[Organization Name] [Athletic Trainer or other medical professional] will be on site for active work areas or in direct communication with management. EMS should be contacted immediately if onsite medical care is not available.

**Exertional Heat Stroke Emergency Procedures (If no cardiac arrest is suspected):**

1. Check the scene
   1. Is it safe for you to help?
   2. What happened?
   3. How many victims are there?
   4. Can bystanders help?
2. Instruct staff member or bystander to call 911

-Provide the following information

1. Who you are
2. General information about the injury or situation
3. Where you are (Provide: name, location of victim, address, telephone #, number of individuals injured, type of injury that has occurred, treatment given, specific directions*). **ADDRESS**

**DIRECTIONS**

**GPS COORDINATES**

1. Any additional information
2. ***STAY ON THE PHONE, BE THE LAST TO HANG UP***
3. Perform emergency cold water immersion (for exertional heat stroke victims)
   1. Fill cold water immersion tub (at least 100-150 gallon tub) up halfway, add ice until water is 35-50°F.
      1. If medical professional is present he/she will stay with victim and provide immediate care.
         1. Have medical provider verify core body temperature
      2. If medical professional is not present, most qualified staff (training/certifications) will stay with victim and provide cooling care.
   2. Initiate cooling (if EHS is suspected and cardiac has been ruled out) – Victim should be secured by 1-2 individuals who may place towels to keep individual above the surface of the water.
      1. Continuously stir water
   3. Continue cooling until :
      1. Body temperature has been reduced to 102°F or lower (confirmed by EMS or onsite medical personnel)
4. Contact the Medical provider for [Organization Name] if not present on scene
5. Meet ambulance and direct to appropriate site
   1. Open Appropriate Gates/Doors
   2. Designate an individual to "flag down" and direct to scene
   3. Control injury site, limit care providers etc.
6. Assist onsite medical provider and/or EMS with care as directed
   1. Retrieve Necessary Supplies/Equipment
7. Document event and debrief

#

# Rehearsal Strategy

*Revise the below statements to match the information provided on page 9 of the “EAP Template Fill-in”. Add, delete, and revise as necessary.*

The [Designated Individual] will be responsible for reviewing the EAP annually and rehearsing it with staff prior to each sport season.

Workers at [Organization Name] will be educated on the EAP prior to their first day and during each year. The meeting will be a requirement for all at risk workers.

The meeting will be directed by [Designated Individual] and will include a power point presentation for recent updates along with a hands-on portion. The hands-on portion will run through different scenarios to ensure the staff understand the EAP. All staff will be provided the opportunity to ask any and all questions and the [Designated Individual] will be responsible for ensuring a proper and adequate answer to all questions.

All staff must sign in to prove their attendance, see following page. The documentation of attendance will be stored with [Designated Individual].

# Documentation of Seasonal Staff Educational Meeting

**Topic: EAP Rehearsal**

**Sign in Sheet**

*This is an example for organizations to utilize for an attendance sheet. Revise or delete as you wish.*

| Name (printed) | Assignment | Signature |
| --- | --- | --- |
|  |  |  |
|  |  |  |
|  |  |  |
|  |  |  |
|  |  |  |
|  |  |  |
|  |  |  |
|  |  |  |
|  |  |  |
|  |  |  |
|  |  |  |
|  |  |  |

Notes:

# Documentation of Emergency Equipment Maintenance

CWI water & ice source

Responsible Party for ensuring Maintenance:

*water and ice source should be checked every month*

| Date | Water/ice source # | Status | Notes |
| --- | --- | --- | --- |
|  |  |  |  |
|  |  |  |  |
|  |  |  |  |
|  |  |  |  |
|  |  |  |  |
|  |  |  |  |
|  |  |  |  |
|  |  |  |  |
|  |  |  |  |
|  |  |  |  |
|  |  |  |  |
|  |  |  |  |
|  |  |  |  |
|  |  |  |  |
|  |  |  |  |
|  |  |  |  |
|  |  |  |  |
|  |  |  |  |
|  |  |  |  |
|  |  |  |  |
|  |  |  |  |
|  |  |  |  |
|  |  |  |  |
|  |  |  |  |

# Approval and Verification Page:

This document has been read and revised by the [Organization Name] manager, supervisor and onsite medical professionals.

**Revise this page based off whom is required to approve the Emergency Action Plan document. Delete this statement prior to use.*

On-Site Manager : _____________________________________ Date:_____________________

Safety Officer : ______________________________________ Date: _____________________

Supervisor : _____________________________________ Date:_____________________

----------------------------------------------------------------------------------------------------------------------------------------------------------------

**The below form is an example form for staff to fill out during the EAP Rehearsal meeting. Revise this form as you see fit for organization, have each staff fill out the form and store this form with the Staff Attendance log from the previous page. Delete this statement prior to use.*

I _____________________________________________________________(print name) have read and understand the Emergency Action Plan for XXXXX. I understand my roles and responsibility should an emergency occur in my presence. I have also rehearsed this Emergency Action Plan and understand my role in an emergency situation with an emergency professionala and without. I have been given the opportunity to ask all questions and have received the proper answers to my questions.

I also understand that I must keep my CPR/AED and First Aid Certifications up to date and that it is my responsibility to ensure a lapse does not occur.

Name (print)_________________________________________

Signature _________________________________________________ Date _________________________

**Appendix E - original version**

**Table S3. SORT Ranking of Citations of the Consensus Document**

| **REFERENCES** | **LOE***  **(1,2,3)** |
| --- | --- |
| ISO 7243:2017. Retrieved March 19, 2021, from ISO website: https://www.iso.org/cms/render/live/en/sites/isoorg/contents/data/standard/06/71/67188.html | **3** |
| (2004). ISO 7933:2004. Retrieved March 19, 2021, from ISO website: https://www.iso.org/cms/render/live/en/sites/isoorg/contents/data/standard/03/76/37600.html | **3** |
| 1910.151—Medical services and first aid. \| Occupational Safety and Health Administration. (1998). Retrieved March 19, 2021, from https://www.osha.gov/laws-regs/regulations/standardnumber/1910/1910.151 | **3** |
| 1926.23—First aid and medical attention. \| Occupational Safety and Health Administration. (n.d.). Retrieved March 19, 2021, from https://www.osha.gov/laws-regs/regulations/standardnumber/1926/1926.23 | **3** |
| ACGIH. (2017). *TLVs and BEIs: Threshold limit values for chemical substances and physical agents and biological exposure indices*. | **3** |
| Adams, W. M., Hosokawa, Y., Huggins, R. A., Mazerolle, S. M., & Casa, D. J. (2016). An Exertional Heat Stroke Survivor’s Return to Running: An Integrated Approach on the Treatment, Recovery, and Return to Activity. *Journal of Sport Rehabilitation*, *25*(3), 280–287. | **3** |
| Adan, A. (2012). Cognitive performance and dehydration. *Journal of the American College of Nutrition*, *31*(2), 71–78. | **3** |
| Aguilar, D. J., & Madero, M. (2019). Other Potential CKD Hotspots in the World: The Cases of Mexico and the United States. *Seminars in Nephrology*, *39*(3), 300–307. | **3** |
| Andersen, J. C., Courson, R. W., Kleiner, D. M., & McLoda, T. A. (2002). National Athletic Trainers’ Association Position Statement: Emergency Planning in Athletics. *Journal of Athletic Training*, *37*(1), 99–104. | **3** |
| Anderson G. Brooke, Bell Michelle L., & Peng Roger D. (2013). Methods to Calculate the Heat Index as an Exposure Metric in Environmental Health Research. *Environmental Health Perspectives*, *121*(10), 1111–1119. | **2** |
| Arbury, S., Lindsley, M., & Hodgson, M. (2016). A Critical Review of OSHA Heat Enforcement Cases: Lessons Learned. *Journal of Occupational and Environmental Medicine*, *58*(4), 359–363. | **3** |
| Armstrong, L. E., & Maresh, C. M. (1991). The induction and decay of heat acclimatisation in trained athletes. *Sports Medicine (Auckland, N.Z.)*, *12*(5), 302–312. | **1** |
| Bach, A. J. E., Maley, M. J., Minett, G. M., Zietek, S. A., Stewart, K. L., & Stewart, I. B. (2019). An Evaluation of Personal Cooling Systems for Reducing Thermal Strain Whilst Working in Chemical/Biological Protective Clothing. *Frontiers in Physiology*, *10*. | **1** |
| Baker, L. B., & Jeukendrup, A. E. (2014). Optimal composition of fluid-replacement beverages. *Comprehensive Physiology*, *4*(2), 575–620. | **2** |
| Belval, L. N., Casa, D. J., Adams, W. M., Chiampas, G. T., Holschen, J. C., Hosokawa, Y., … Stearns, R. L. (2018). Consensus Statement- Prehospital Care of Exertional Heat Stroke. *Prehospital Emergency Care*, *22*(3), 392–397. | **3** |
| Benjamin, C. L., Sekiguchi, Y., Fry, L. A., & Casa, D. J. (2019). Performance Changes Following Heat Acclimation and the Factors That Influence These Changes: Meta-Analysis and Meta-Regression. *Frontiers in Physiology*, *10*, 1448. | **1** |
| Bernard, T. E., Caravello, V., Schwartz, S. W., & Ashley, C. D. (2008). WBGT clothing adjustment factors for four clothing ensembles and the effects of metabolic demands. *Journal of Occupational and Environmental Hygiene*, *5*(1), 1–5; quiz d21-23. | **1** |
| Bernard, T. E., & Iheanacho, I. (2015). Heat index and adjusted temperature as surrogates for wet bulb globe temperature to screen for occupational heat stress. *Journal of Occupational and Environmental Hygiene*, *12*(5), 323–333. | **1** |
| Bernard, T. E., Luecke, C. L., Schwartz, S. K., Kirkland, K. S., & Ashley, C. D. (2005). WBGT Clothing Adjustments for Four Clothing Ensembles Under Three Relative Humidity Levels. *Journal of Occupational and Environmental Hygiene*, *2*(5), 251–256. | **1** |
| Bethancourt, H. J., Swanson, Z. S., Nzunza, R., Huanca, T., Conde, E., Kenney, W. L., … Rosinger, A. Y. (2021). Hydration in relation to water insecurity, heat index, and lactation status in two small-scale populations in hot-humid and hot-arid environments. *American Journal of Human Biology: The Official Journal of the Human Biology Council*, *33*(1), e23447. | **2** |
| Biggs, C., Paterson, M., & Maunder, E. (2011). Hydration status of South African forestry workers harvesting trees in autumn and winter. *The Annals of Occupational Hygiene*, *55*(1), 6–15. | **2** |
| Błażejczyk, K., Jendritzky, G., Bröde, P., Fiala, D., Havenith, G., Epstein, Y., ... & Kampmann, B. (2013). An introduction to the universal thermal climate index (UTCI). *Geographia Polonica*, *86*(1), 5-10 | **3** |
| Bongers, C. C. W. G., Hopman, M. T. E., & Eijsvogels, T. M. H. (2017). Cooling interventions for athletes: An overview of effectiveness, physiological mechanisms, and practical considerations. *Temperature: Multidisciplinary Biomedical Journal*, *4*(1), 60–78. | **3** |
| Bourlai, T., Pryor, R. R., Suyama, J., Reis, S. E., & Hostler, D. (2012). Use of thermal imagery for estimation of core body temperature during precooling, exertion, and recovery in wildland firefighter protective clothing. *Prehospital Emergency Care: Official Journal of the National Association of EMS Physicians and the National Association of State EMS Directors*, *16*(3), 390–399. | **3** |
| Bouskill, L. M. (1999). *Clothing ventilation and human thermal response* (Ph.D., Loughborough University). Loughborough University. Retrieved from https://hdl.handle.net/2134/7055 | **3** |
| Brake, D. J., & Bates, G. P. (2003). Fluid losses and hydration status of industrial workers under thermal stress working extended shifts. *Occupational and Environmental Medicine*, *60*(2), 90–96. | **3** |
| Brearley, M., Harrington, P., Lee, D., & Taylor, R. (2015). Working in hot conditions—A study of electrical utility workers in the northern territory of Australia. *Journal of Occupational and Environmental Hygiene*, *12*(3), 156–162. | **3** |
| Brearley, M., & Walker, A. (2015). Water immersion for post incident cooling of firefighters; a review of practical fire ground cooling modalities. *Extreme Physiology & Medicine*, *4*. | **3** |
| Budd, G. M. (2008). Wet-bulb globe temperature (WBGT)—Its history and its limitations. *Journal of Science and Medicine in Sport*, *11*(1), 20–32. | **3** |
| Buller, M. J., Tharion, W. J., Cheuvront, S. N., Montain, S. J., Kenefick, R. W., Castellani, J., … Hoyt, R. W. (2013). Estimation of human core temperature from sequential heart rate observations. *Physiological Measurement*, *34*(7), 781–798. | **3** |
| Butler-Dawson, J., Krisher, L., Yoder, H., Dally, M., Sorensen, C., Johnson, R. J., … Newman, L. S. (2019). Evaluation of heat stress and cumulative incidence of acute kidney injury in sugarcane workers in Guatemala. *International Archives of Occupational and Environmental Health*, *92*(7), 977–990. | **3** |
| Butts, C. L., Smith, C. R., Ganio, M. S., & McDermott, B. P. (2017). Physiological and perceptual effects of a cooling garment during simulated industrial work in the heat. *Applied Ergonomics*, *59*, 442–448. | **1** |
| Calculating Workplace WBGT from Meteorological Data: A Tool for Climate Change Assessment \| Climate CHIP. (2012). Retrieved March 19, 2021, from https://www.climatechip.org/node/56 | **3** |
| Carter, M. R., McGinn, R., Barrera-Ramirez, J., Sigal, R. J., & Kenny, G. P. (2014). Impairments in local heat loss in type 1 diabetes during exercise in the heat. *Medicine and Science in Sports and Exercise*, *46*(12), 2224–2233. | **1** |
| Casa, D. J., Anderson, S. A., Baker, L., Bennett, S., Bergeron, M. F., Connolly, D., … Thompson, C. (2012). The Inter-Association Task Force for Preventing Sudden Death in Collegiate Conditioning Sessions: Best Practices Recommendations. *Journal of Athletic Training*, *47*(4), 477–480. | **3** |
| Casa, D. J., DeMartini, J. K., Bergeron, M. F., Csillan, D., Eichner, E. R., Lopez, R. M., … Yeargin, S. W. (2015). National Athletic Trainers’ Association Position Statement: Exertional Heat Illnesses. *Journal of Athletic Training*, *50*(9), 986–1000. | **3** |
| Casa, D. J., McDermott, B. P., Lee, E. C., Yeargin, S. W., Armstrong, L. E., & Maresh, C. M. (2007). Cold water immersion: The gold standard for exertional heatstroke treatment. *Exercise and Sport Sciences Reviews*, *35*(3), 141–149. | **3** |
| CFOI. (2018). Census of Fatal Occupational Injuries (CFOI)—Current and Revised Data. Retrieved May 6, 2020, from https://www.bls.gov/iif/oshcfoi1.htm#2018 | **N/A** |
| Cheung, S. S. (2010). Interconnections between thermal perception and exercise capacity in the heat. *Scandinavian Journal of Medicine & Science in Sports*, *20*(s3), 53–59. | **3** |
| Cheuvront, S. N., & Kenefick, R. W. (2014). Dehydration: Physiology, assessment, and performance effects. *Comprehensive Physiology*, *4*(1), 257–285. | **2** |
| Cheuvront, S. N., & Kenefick, R. W. (2016). Am I Drinking Enough? Yes, No, and Maybe. *Journal of the American College of Nutrition*, *35*(2), 185–192. | **3** |
| Chicas, R., Xiuhtecutli, N., Dickman, N. E., Scammell, M. L., Steenland, K., Hertzberg, V. S., & McCauley, L. (2020). Cooling intervention studies among outdoor occupational groups: A review of the literature. *American Journal of Industrial Medicine*, *63*(11), 988–1007. | **3** |
| Clapp, A. J., Bishop, P. A., Smith, J. F., & Mansfield, E. R. (2000). Effects of carbohydrate-electrolyte content of beverages on voluntary hydration in a simulated industrial environment. *AIHAJ: A Journal for the Science of Occupational and Environmental Health and Safety*, *61*(5), 692–699. | **3** |
| Clapp, A. J., Bishop, P. A., & Walker, J. L. (1999). Fluid replacement preferences in heat-exposed workers. *American Industrial Hygiene Association Journal*, *60*(6), 747–751. | **3** |
| Comparative Assessment of GORETEX^TM^ and NEOPRENE^TM^ Vapor Barriers in a Firefighter Turn-Out Coat—Uwe Reischl, Alfred Stransky, 1980. (n.d.). Retrieved March 19, 2021, from https://journals.sagepub.com/doi/10.1177/004051758005001101 | **3** |
| Cooper, E., Grundstein, A., Rosen, A., Miles, J., Ko, J., & Curry, P. (2017). An Evaluation of Portable Wet Bulb Globe Temperature Monitor Accuracy. *Journal of Athletic Training*, *52*(12), 1161–1167. | **3** |
| Courson, R. (2007). Preventing sudden death on the athletic field: The emergency action plan. *Current Sports Medicine Reports*, *6*(2), 93–100. | **2** |
| Cuddy, J. S., & Ruby, B. C. (2011). High work output combined with high ambient temperatures caused heat exhaustion in a wildland firefighter despite high fluid intake. *Wilderness & Environmental Medicine*, *22*(2), 122–125. | **3** |
| Daanen, H. A. M., Racinais, S., & Périard, J. D. (2018). Heat Acclimation Decay and Re-Induction: A Systematic Review and Meta-Analysis. *Sports Medicine (Auckland, N.Z.)*, *48*(2), 409–430. | **1** |
| Davison, R. R., Van Someren, K. A., & Jones, A. M. (2009). Physiological monitoring of the Olympic athlete. *Journal of Sports Sciences*, *27*(13), 1433–1442. | **3** |
| DeMartini, J. K., Casa, D. J., Stearns, R., Belval, L., Crago, A., Davis, R., & Jardine, J. (2015). Effectiveness of cold water immersion in the treatment of exertional heat stroke at the Falmouth Road Race. *Medicine and Science in Sports and Exercise*, *47*(2), 240–245. | **3** |
| DeMartini, J. K., Ranalli, G. F., Casa, D. J., Lopez, R. M., Ganio, M. S., Stearns, R. L., … Maresh, C. M. (2011). Comparison of body cooling methods on physiological and perceptual measures of mildly hyperthermic athletes. *Journal of Strength and Conditioning Research*, *25*(8), 2065–2074. | **3** |
| Dervis, S., Coombs, G. B., Chaseling, G. K., Filingeri, D., Smoljanic, J., & Jay, O. (2016). A comparison of thermoregulatory responses to exercise between mass-matched groups with large differences in body fat. *Journal of Applied Physiology (Bethesda, Md.: 1985)*, *120*(6), 615–623. | **3** |
| Drezner, J. A., Toresdahl, B. G., Rao, A. L., Huszti, E., & Harmon, K. G. (2013). Outcomes from sudden cardiac arrest in US high schools: A 2-year prospective study from the National Registry for AED Use in Sports. *British Journal of Sports Medicine*, *47*(18), 1179–1183. | **3** |
| Drezner Jonathan A., Rao Ashwin L., Heistand Justin, Bloomingdale Megan K., & Harmon Kimberly G. (2009). Effectiveness of Emergency Response Planning for Sudden Cardiac Arrest in United States High Schools With Automated External Defibrillators. *Circulation*, *120*(6), 518–525. | **3** |
| Ebell, M. H., Siwek, J., Weiss, B. D., Woolf, S. H., Susman, J., Ewigman, B., & Bowman, M. A. (2004). Strength of Recommendation Taxonomy (SORT): A Patient-Centered Approach to Grading Evidence in the Medical Literature. *American Family Physician*, *69*(3), 548. | **N/A** |
| El-Shafei, D. A., Bolbol, S. A., Awad Allah, M. B., & Abdelsalam, A. E. (2018). Exertional heat illness: Knowledge and behavior among construction workers. *Environmental Science and Pollution Research*, *25*(32), 32269–32276. | **3** |
| Epstein, Y., & Yanovich, R. (2019). Heatstroke. *The New England Journal of Medicine*, *380*(25), 2449–2459. | **2** |
| Flouris, A. D., Dinas, P. C., Ioannou, L. G., Nybo, L., Havenith, G., Kenny, G. P., & Kjellstrom, T. (2018). Workers’ health and productivity under occupational heat strain: A systematic review and meta-analysis. *The Lancet Planetary Health*, *2*(12), e521–e531. | **3** |
| Foster, J., Hodder, S. G., Goodwin, J., & Havenith, G. (2020). Occupational Heat Stress and Practical Cooling Solutions for Healthcare and Industry Workers During the COVID-19 Pandemic. *Annals of Work Exposures and Health*, *64*(9), 915–922. | **3** |
| Frank, A., Belokopytov, M., Shapiro, Y., & Epstein, Y. (2001). The cumulative heat strain index—A novel approach to assess the physiological strain induced by exercise-heat stress. *European Journal of Applied Physiology*, *84*(6), 527–532. | **3** |
| Friedl, K. E. (2018). Military applications of soldier physiological monitoring. *Journal of Science and Medicine in Sport*, *21*(11), 1147–1153. | **3** |
| McQuerry, M., Barker, R., & DenHartog, E. (2018). Functional Design and Evaluation of Structural Firefighter Turnout Suits for Improved Thermal Comfort: Thermal Manikin and Physiological Modeling. *Clothing and Textiles Research Journal*, *36*(3), 165-179. | **3** |
| Ganio, M. S., Armstrong, L. E., Casa, D. J., McDermott, B. P., Lee, E. C., Yamamoto, L. M., ... & Lieberman, H. R. (2011). Mild dehydration impairs cognitive performance and mood of men. *British Journal of Nutrition*, *106*(10), 1535-1543. | **1** |
| Glaser, J., Lemery, J., Rajagopalan, B., Diaz, H. F., García-Trabanino, R., Taduri, G., … Johnson, R. J. (2016). Climate Change and the Emergent Epidemic of CKD from Heat Stress in Rural Communities: The Case for Heat Stress Nephropathy. *Clinical Journal of the American Society of Nephrology: CJASN*, *11*(8), 1472–1483. | **3** |
| Greenleaf, J. E. (1992). Problem: Thirst, drinking behavior, and involuntary dehydration. *Medicine and Science in Sports and Exercise*, *24*(6), 645–656. | **2** |
| Grundstein, A., & Cooper, E. (2020). Comparison of WBGTs over Different Surfaces within an Athletic Complex. *Medicina*, *56*(6). | **1** |
| Gun, R. T., & Budd, G. M. (1995). Effects of thermal, personal and behavioural factors on the physiological strain, thermal comfort and productivity of Australian shearers in hot weather. *Ergonomics*, *38*(7), 1368–1384. | **3** |
| Hansson, E., Glaser, J., Jakobsson, K., Weiss, I., Wesseling, C., Lucas, R. A. I., … Wegman, D. H. (2020). Pathophysiological Mechanisms by which Heat Stress Potentially Induces Kidney Inflammation and Chronic Kidney Disease in Sugarcane Workers. *Nutrients*, *12*(6). | **3** |
| Havenith, G. (1999). Heat balance when wearing protective clothing. *The Annals of Occupational Hygiene*, *43*(5), 289–296. | **3** |
| Havenith, G., Heus, R., & Lotens, W. A. (1990). Resultant clothing insulation: A function of body movement, posture, wind, clothing fit and ensemble thickness. *Ergonomics*, *33*(1), 67–84. | **3** |
| He, J., Lu, Y., Wang, L., & Ma, N. (2018). On the Improvement of Thermal Protection for Temperature-Responsive Protective Clothing Incorporated with Shape Memory Alloy. *Materials (Basel, Switzerland)*, *11*(10). | **3** |
| Heat—OSHA Outdoor WBGT Calculator \| Occupational Safety and Health Administration. (n.d.). Retrieved March 19, 2021, from <https://www.osha.gov/heat-exposure/wbgt-calculator> | **3** |
| Holm, C. A., Pahler, L., Thiese, M. S., & Handy, R. (2016). Evaluation of physiological strain in hot work areas using thermal imagery. *Journal of Thermal Biology*, *61*, 8–15. | **3** |
| Horn, G. P., Gutzmer, S., Fahs, C. A., Petruzzello, S. J., Goldstein, E., Fahey, G. C., … Smith, D. L. (2011). Physiological recovery from firefighting activities in rehabilitation and beyond. *Prehospital Emergency Care: Official Journal of the National Association of EMS Physicians and the National Association of State EMS Directors*, *15*(2), 214–225. | **3** |
| Hosokawa, Y. (2019). Activity modification in heat: Critical assessment of guidelines across athletic, occupational, and military settings in the USA \| SpringerLink. Retrieved September 15, 2019, from <https://link-springer-com.ezproxy.lib.uconn.edu/article/10.1007/s00484-019-01673-6> | **3** |
| Hospers, L., Smallcombe, J. W., Morris, N. B., Capon, A., & Jay, O. (2020). Electric fans: A potential stay-at-home cooling strategy during the COVID-19 pandemic this summer? *The Science of the Total Environment*, *747*, 141180. | **3** |
| Hunt, A. P., Billing, D. C., Patterson, M. J., & Caldwell, J. N. (2016). Heat strain during military training activities: The dilemma of balancing force protection and operational capability. *Temperature (Austin, Tex.)*, *3*(2), 307–317. | **3** |
| Hygiene. (n.d.). Retrieved March 18, 2021, from WHO \| Regional Office for Africa website: <https://www.afro.who.int/health-topics/hygiene> | **N/A** |
| Jacklitsch, B. L., King, K. A., Vidourek, R. A., & Merianos, A. L. (2018). Heat-Related Training and Educational Material Needs among Oil Spill Cleanup Responders. *Environmental Health Insights*, *12*, 1178630218802295. | **3** |
| Jacklitsch B, Williams WJ, Musolin K, Coca A, Kim J-H, Turner. (2016). *Criteria for a recommended standard: Occupational exposure to heat and hot environments - revised criteria 2016.* | **3** |
| Jin, L., Cao, M. L., Yu, W., Hu, J. Y., Yoon, K. J., Park, P. K., & Li, Y. (2018). New Approaches to Evaluate the Performance of Firefighter Protective Clothing Materials. *Fire Technology*, *54*(5), 1283–1307. | **3** |
| Johnson, R. J., Wesseling, C., & Newman, L. S. (2019). Chronic Kidney Disease of Unknown Cause in Agricultural Communities. *The New England Journal of Medicine*, *380*(19), 1843–1852. | **3** |
| Jones, P. R., Barton, C., Morrissey, D., Maffulli, N., & Hemmings, S. (2012). Pre-cooling for endurance exercise performance in the heat: A systematic review. *BMC Medicine*, *10*(1), 166. | **3** |
| Kavouras, S. A. (2019). Hydration, dehydration, underhydration, optimal hydration: Are we barking up the wrong tree? *European Journal of Nutrition*, *58*(2), 471–473. | **3** |
| Kenefick, R. W., & Sawka, M. N. (2007). Hydration at the work site. *Journal of the American College of Nutrition*, *26*(5 Suppl), 597S-603S. | **3** |
| Kenny, G. P., Stapleton, J. M., Yardley, J. E., Boulay, P., & Sigal, R. J. (2013). Older adults with type 2 diabetes store more heat during exercise. *Medicine and Science in Sports and Exercise*, *45*(10), 1906–1914. | **3** |
| Kenny, G. P., Yardley, J., Brown, C., Sigal, R. J., & Jay, O. (2010). Heat stress in older individuals and patients with common chronic diseases. *CMAJ: Canadian Medical Association Journal = Journal de l’Association Medicale Canadienne*, *182*(10), 1053– | **3** |
| Kerr, Z. Y., Register-Mihalik, J. K., Pryor, R. R., Pierpoint, L. A., Scarneo, S. E., Adams, W. M., … Marshall, S. W. (2019). The Association between Mandated Preseason Heat Acclimatization Guidelines and Exertional Heat Illness during Preseason High School American Football Practices. *Environmental Health Perspectives*, *127*(4), 47003. | **3** |
| Kiely, M., Warrington, G., McGoldrick, A., & Cullen, S. (2019). Physiological and Performance Monitoring in Competitive Sporting Environments: A Review for Elite Individual Sports. *Strength & Conditioning Journal*, *41*(6), 62–74. | **3** |
| Kim, S., Kim, D.-H., Lee, H.-H., & Lee, J.-Y. (2019). Frequency of firefighters’ heat-related illness and its association with removing personal protective equipment and working hours. *Industrial Health*, *57*(3), 370–380. | **3** |
| Kjellstrom, T., Briggs, D., Freyberg, C., Lemke, B., Otto, M., & Hyatt, O. (2016). *Heat, Human Performance, and Occupational Health: A Key Issue for the Assessment of Global Climate Change Impacts.* | **2** |
| Kjellstrom, Tord, Holmer, I., & Lemke, B. (2009). Workplace heat stress, health and productivity – an increasing challenge for low and middle-income countries during climate change. *Global Health Action*, *2*(1), 2047. | **3** |
| Kjellstrom, T., Lemke, B., Otto, M., Hyatt, O., Briggs, D., & Freyberg, C. (2014). Threats to occupational health, labor productivity and the economy from increasing heat during climate change: an emerging global health risk and a challenge to sustainable development and social equity. *Mapua: Health and Environment International Trust*. | **3** |
| Kroshus, E., Wagner, J., Wyrick, D., Athey, A., Bell, L., Benjamin, H. J., … Hainline, B. (2019). Wake up call for collegiate athlete sleep: Narrative review and consensus recommendations from the NCAA Interassociation Task Force on Sleep and Wellness. *British Journal of Sports Medicine*, *53*(12), 731–736. | **3** |
| Lee, S. W., Lee, K., & Lim, B. (2018). Effects of climate change-related heat stress on labor productivity in South Korea. *International journal of biometeorology*, *62*(12), 2119-2129. | **3** |
| Liljegren, J. C., Carhart, R. A., Lawday, P., Tschopp, S., & Sharp, R. (2008). Modeling the wet bulb globe temperature using standard meteorological measurements. *Journal of Occupational and Environmental Hygiene*, *5*(10), 645–655. | **3** |
| Lucas, R. A. I., Ganio, M. S., Pearson, J., & Crandall, C. G. (2013). Sweat loss during heat stress contributes to subsequent reductions in lower-body negative pressure tolerance. *Experimental Physiology*, *98*(2), 473–480. | **3** |
| Lumley, S. H., Story, D. L., & Thomas, N. T. (1991). Clothing ventilation—Update and applications. *Applied Ergonomics*, *22*(6), 390–394. | **3** |
| Mansor, Z., Rosnah, I., Ismail, N. H., & Hashim, J. H. (2019). Effects of hydration practices on the severity of heat-related illness among municipal workers during a heat wave phenomenon. *The Medical Journal of Malaysia*, *74*(4), 275–280. | **3** |
| McCarthy, R. B., Shofer, F. S., & Green-McKenzie, J. (2019). Outcomes of a Heat Stress Awareness Program on Heat-Related Illness in Municipal Outdoor Workers. *Journal of Occupational and Environmental Medicine*, *61*(9), 724–728. | **3** |
| McDermott, B. P., Casa, D. J., Ganio, M. S., Lopez, R. M., Yeargin, S. W., Armstrong, L. E., & Maresh, C. M. (2009). Acute Whole-Body Cooling for Exercise-Induced Hyperthermia: A Systematic Review. *Journal of Athletic Training*, *44*(1), 84–93. | **3** |
| McDermott, B. P., Casa, D. J., Yeargin, S. W., Ganio, M. S., Armstrong, L. E., & Maresh, C. M. (2007). Recovery and return to activity following exertional heat stroke: Considerations for the sports medicine staff. *Journal of Sport Rehabilitation*, *16*(3), 163–181. | **3** |
| McEntire, S. J., Suyama, J., & Hostler, D. (2013). Mitigation and prevention of exertional heat stress in firefighters: A review of cooling strategies for structural firefighting and hazardous materials responders. *Prehospital Emergency Care: Official Journal of the National Association of EMS Physicians and the National Association of State EMS Directors*, *17*(2), 241–260. | **3** |
| McFarlin, B. K., Henning, A. L., Venable, A. S., Williams, R. R., & Best Sampson, J. N. (2016). A shirt containing multistage phase change material and active cooling components was associated with increased exercise capacity in a hot, humid environment. *Ergonomics*, *59*(8), 1019–1025. | **3** |
| McLellan, T. M., & Havenith, G. (2016). Protective clothing ensembles and physical employment standards. *Applied Physiology, Nutrition, and Metabolism = Physiologie Appliquee, Nutrition Et Metabolisme*, *41*(6 Suppl 2), S121-130. | **3** |
| McQuerry, M., Barker, R., & DenHartog, E. (2018). Relationship between novel design modifications and heat stress relief in structural firefighters’ protective clothing. *Applied Ergonomics*, *70*, 260–268. | **3** |
| McQuerry, M., DenHartog, E., & Barker, R. (2018). Impact of reinforcements on heat stress in structural firefighter turnout suits. *The Journal of The Textile Institute*, *109*(10), 1367–1373. | **3** |
| McQuerry, M., Morrissey, M., Kisiolek, J., Gipson, S., Ormsbee, M., & Ormsbee, M. (2020). Effect of a Lightweight Structural Firefighter Turnout Composite on Physiological Comfort. *Performance of Protective Clothing and Equipment: 11th Volume, Innovative Solutions to Evolving Challenges*. | **1** |
| Mekonnen, M. M., & Hoekstra, A. Y. (2016). Four billion people facing severe water scarcity. *Science Advances*, *2*(2), e1500323. | **3** |
| Miller, V. S., & Bates, G. P. (2010). Hydration, hydration, hydration. *The Annals of Occupational Hygiene*, *54*(2), 134–136. | **3** |
| Mix, J., Elon, L., Vi Thien Mac, V., Flocks, J., Economos, E., Tovar-Aguilar, A. J., … McCauley, L. A. (2018). Hydration Status, Kidney Function, and Kidney Injury in Florida Agricultural Workers. *Journal of Occupational and Environmental Medicine*, *60*(5), e253–e260. | **3** |
| Moda, H. M., Filho, W. L., & Minhas, A. (2019). Impacts of Climate Change on Outdoor Workers and Their Safety: Some Research Priorities. *International Journal of Environmental Research and Public Health*, *16*(18). | **3** |
| Moran, D. S., Shitzer, A., & Pandolf, K. B. (1998). A physiological strain index to evaluate heat stress. *The American Journal of Physiology*, *275*(1), R129-134. | **2** |
| Morris, N. B., Coombs, G., & Jay, O. (2016). Ice Slurry Ingestion Leads to a Lower Net Heat Loss during Exercise in the Heat. *Medicine and Science in Sports and Exercise*, *48*(1), 114–122. | **1** |
| Morris, N. B., Jay, O., Flouris, A. D., Casanueva, A., Gao, C., Foster, J., … Nybo, L. (2020). Sustainable solutions to mitigate occupational heat strain – an umbrella review of physiological effects and global health perspectives. *Environmental Health*, *19*. | **3** |
| Morris, N. B., Levi, M., Morabito, M., Messeri, A., Ioannou, L. G., Flouris, A. D., … Nybo, L. (2020). Health vs. wealth: Employer, employee and policy-maker perspectives on occupational heat stress across multiple European industries. *Temperature*, *0*(0), 1–18. | **3** |
| Moseley, P. L. (1994). Mechanisms of heat adaptation: Thermotolerance and acclimatization. *The Journal of Laboratory and Clinical Medicine*, *123*(1), 48–52. | **3** |
| Nerbass, F. B., Moist, L., Clark, W. F., Vieira, M. A., & Pecoits-Filho, R. (2019). Hydration Status and Kidney Health of Factory Workers Exposed to Heat Stress: A Pilot Feasibility Study. *Annals of Nutrition & Metabolism*, *74 Suppl 3*, 30–37. | **3** |
| Nerbass, F. B., Pecoits-Filho, R., Clark, W. F., Sontrop, J. M., McIntyre, C. W., & Moist, L. (2017). Occupational Heat Stress and Kidney Health: From Farms to Factories. *Kidney International Reports*, *2*(6), 998–1008. | **3** |
| NFPA 1584: Standard on the Rehabilitation Process for Members During Emergency Operations and Training Exercises. (2015). Retrieved March 19, 2021, from https://www.nfpa.org/codes-and-standards/all-codes-and-standards/list-of-codes-and-standards/detail?code=1584 | **3** |
| NOAA’s National Weather Service—Graphical Forecast. (n.d.). Retrieved March 19, 2021, from https://graphical.weather.gov/ | **3** |
| Notley, S. R., Flouris, A. D., & Kenny, G. P. (2018). On the use of wearable physiological monitors to assess heat strain during occupational heat stress. *Applied Physiology, Nutrition, and Metabolism = Physiologie Appliquee, Nutrition Et Metabolisme*, *43*(9), 869–881. | **2** |
| Notley, S. R., Poirier, M. P., Sigal, R. J., D’Souza, A., Flouris, A. D., Fujii, N., & Kenny, G. P. (2019). Exercise Heat Stress in Patients With and Without Type 2 Diabetes. *JAMA*, *322*(14), 1409–1411. | **1** |
| Nunfam, V. F., Adusei-Asante, K., Van Etten, E. J., Oosthuizen, J., & Frimpong, K. (2018). Social impacts of occupational heat stress and adaptation strategies of workers: A narrative synthesis of the literature. *Science of The Total Environment*, *643*, 1542–1552. | **1** |
| Occupational Hygiene—IOHA. Accessed October 16, 2020. Https://www.ioha.net/about/occupational-hygiene/—Google Search. (n.d.). Retrieved March 18, 2021, from https://www.google.com/search?q=Occupational+Hygiene+-+IOHA.+Accessed+October+16%2C+2020.+https%3A%2F%2Fwww.ioha.net%2Fabout%2Foccupational-hygiene%2F&rlz=1C5CHFA_enUS937US938&oq=Occupational+Hygiene+-+IOHA.+Accessed+October+16%2C+2020.+https%3A%2F%2Fwww.ioha.net%2Fabout%2Foccupational-hygiene%2F&aqs=chrome..69i57.1235j0j4&sourceid=chrome&ie=UTF-8 | **N/A** |
| OSH Act of 1970 \| Occupational Safety and Health Administration. (1970). Retrieved March 19, 2021, from https://www.osha.gov/laws-regs/oshact/completeoshact | **N/A** |
| OSHA’s Campaign to Prevent Heat Illness in Outdoor Workers \| Heat Fatalities [Text Version] \| Occupational Safety and Health Administration. (2011). Retrieved September 13, 2020, from https://www.osha.gov/SLTC/heatillness/map_text.html | **3** |
| Pandolf, K. B., & Goldman, R. F. (1978). Convergence of skin and rectal temperatures as a criterion for heat tolerance. *Aviation, Space, and Environmental Medicine*, *49*(9), 1095–1101. | **3** |
| Park, Jungsun, Kim, Y., & Oh, I. (2017). Factors affecting heat-related diseases in outdoor workers exposed to extreme heat. *Annals of Occupational and Environmental Medicine*, *29*, 30. | **3** |
| Park, Juyeon, & Langseth-Schmidt, K. (2016). Anthropometric fit evaluation of firefighters’ uniform pants: A sex comparison. *International Journal of Industrial Ergonomics*, *56*, 1–8. | **2** |
| Parsons, K. (2009). Maintaining health, comfort and productivity in heat waves. *Global Health Action*, *2*. | **2** |
| Périard, J. D., Racinais, S., & Sawka, M. N. (2015). Adaptations and mechanisms of human heat acclimation: Applications for competitive athletes and sports. *Scandinavian Journal of Medicine & Science in Sports*, *25 Suppl 1*, 20–38. | **3** |
| Pescatello, L. S., Mack, G. W., Leach, C. N., & Nadel, E. R. (1987). Effect of beta-adrenergic blockade on thermoregulation during exercise. *Journal of Applied Physiology*, *62*(4), 1448–1452. | **3** |
| Piil, J. F., Lundbye-Jensen, J., Christiansen, L., Ioannou, L., Tsoutsoubi, L., Dallas, C. N., … Nybo, L. (2018). High prevalence of hypohydration in occupations with heat stress-perspectives for performance in combined cognitive and motor tasks. *PloS One*, *13*(10), e0205321. | **2** |
| Price, K., Benmarhnia, T., Gaudet, J., Kaiser, D., Sadoine, M. L., Perron, S., & Smargiassi, A. (2018). The Montreal heat response plan: Evaluation of its implementation towards healthcare professionals and vulnerable populations. *Canadian Journal of Public Health = Revue Canadienne De Sante Publique*, *109*(1), 108–116. | **3** |
| Progress on drinking water, sanitation and hygiene in schools \| UNICEF. (2020). Retrieved March 18, 2021, from https://www.unicef.org/reports/progress-on-drinking-water-sanitation-and-hygiene-in-schools-focus-on-covid-19 | **3** |
| Pryor, J. L., Pryor, R. R., Vandermark, L. W., Adams, E. L., VanScoy, R. M., Casa, D. J., … Maresh, C. M. (2019). Intermittent exercise-heat exposures and intense physical activity sustain heat acclimation adaptations. *Journal of Science and Medicine in Sport*, *22*(1), 117–122. | **2** |
| Pryor, R. R., Pryor, J. L., Vandermark, L. W., Adams, E. L., Brodeur, R. M., Armstrong, L. E., … Casa, D. J. (2019). Exacerbated heat strain during consecutive days of repeated exercise sessions in heat. *Journal of Science and Medicine in Sport*, *22*(10), 1084–1089. | **1** |
| Psikuta, A., Koelblen, B., Mert, E., Fontana, P., & Annaheim, S. (2017). An integrated approach to develop, validate and operate thermo-physiological human simulator for the development of protective clothing. *Industrial Health*, *55*(6), 500–512. | **3** |
| Puga, A. M., Lopez-Oliva, S., Trives, C., Partearroyo, T., & Varela-Moreiras, G. (2019). Effects of Drugs and Excipients on Hydration Status. *Nutrients*, *11*(3). | **1** |
| Ribeiro, G. A., Rodrigues, L. O. C., Moreira, M. C. V., Silami-Garcia, E., Pascoa, M. R. S., & Camargos, F. F. O. (2004). Thermoregulation in hypertensive men exercising in the heat with water ingestion. *Brazilian Journal of Medical and Biological Research*, *37*(3), 409–417. | **2** |
| Riley, K., Delp, L., Cornelio, D., & Jacobs, S. (2012). From agricultural fields to urban asphalt: The role of worker education to promote California’s heat illness prevention standard. *New Solutions: A Journal of Environmental and Occupational Health Policy: NS*, *22*(3), 297–323. | **2** |
| Rosinger, A. Y. (2018). Household water insecurity after a historic flood: Diarrhea and dehydration in the Bolivian Amazon. *Social Science & Medicine (1982)*, *197*, 192–202. | **3** |
| Rothfusz, L. P. (1990). *The Heat Index “Equation” (or, More Than You Ever Wanted to Know About Heat Index)*. 2. | **3** |
| Scarneo, S. E., DiStefano, L. J., Stearns, R. L., Register-Mihalik, J. K., Denegar, C. R., & Casa, D. J. (2019). Emergency Action Planning in Secondary School Athletics: A Comprehensive Evaluation of Current Adoption of Best Practice Standards. *Journal of Athletic Training*, *54*(1), 99–105. |  |
| Scarneo-Miller, S. E., Kerr, Z. Y., Adams, W. M., Belval, L. N., & Casa, D. J. (2020). Influence of State-Level Emergency Planning Policy Requirements on Secondary School Adoption. *Journal of Athletic Training*, *55*(10), 1062–1069. | **2** |
| Schlader, Z. J., Colburn, D., & Hostler, D. (2017). Heat Strain Is Exacerbated on the Second of Consecutive Days of Fire Suppression. *Medicine and Science in Sports and Exercise*, *49*(5), 999–1005. | **1** |
| Schlader, Z. J., Gagnon, D., Rivas, E., Convertino, V. A., & Crandall, C. G. (2015). Fluid restriction during exercise in the heat reduces tolerance to progressive central hypovolaemia. *Experimental Physiology*, *100*(8), 926–934. | **1** |
| Sekiguchi, Y., Filep, E. M., Benjamin, C. L., Casa, D. J., & DiStefano, L. J. (2020). Does Dehydration Affect the Adaptations of Plasma Volume, Heart Rate, Internal Body Temperature, and Sweat Rate During the Induction Phase of Heat Acclimation? *Journal of Sport Rehabilitation*, *29*(6), 847–850. | **3** |
| Sinclair, R. C., & Cunningham, T. R. (2014). Safety activities in small businesses. *Safety Science*, *64*, 32–38. | **3** |
| Song, W., & Wang, F. (2016). The hybrid personal cooling system (PCS) could effectively reduce the heat strain while exercising in a hot and moderate humid environment. *Ergonomics*, *59*(8), 1009-1018. | **2** |
| Spector, J. T., Masuda, Y. J., Wolff, N. H., Calkins, M., & Seixas, N. (2019). Heat Exposure and Occupational Injuries: Review of the Literature and Implications. *Current Environmental Health Reports*, *6*(4), 286–296. | **2** |
| Stearns, R. L., Casa, D. J., O’Connor, F. G., & Lopez, R. M. (2016). A Tale of Two Heat Strokes: A Comparative Case Study. *Current Sports Medicine Reports*, *15*(2), 94–97. | **3** |
| Tanglis, M. (2018, July 17). It is Time to Protect Millions of Workers From Extreme Heat. Retrieved April 6, 2021, from CitizenVox website: https://citizenvox.org/2018/07/17/workers-in-extreme-heat/ | **3** |
| The physiological equivalent temperature—A universal index for the biometeorological assessment of the thermal environment—PubMed. (1999). Retrieved April 13, 2021, from https://pubmed.ncbi.nlm.nih.gov/10552310/ | **3** |
| Tokizawa, K., Son, S.-Y., Oka, T., & Yasuda, A. (2020). Effectiveness of a field-type liquid cooling vest for reducing heat strain while wearing protective clothing. *Industrial Health*, *58*(1), 63–71. | **1** |
| Travers, G. J. S., Nichols, D. S., Farooq, A., Racinais, S., & Périard, J. D. (2016). Validation of an ingestible temperature data logging and telemetry system during exercise in the heat. *Temperature*, *3*(2), 208–219. | **3** |
| Trites, D. G., Robinson, D. G., & Banister, E. W. (1993). Cardiovascular and muscular strain during a tree planting season among British Columbia silviculture workers. *Ergonomics*, *36*(8), 935–949. | **3** |
| Tucker, M. A., Caldwell, A. R., Butts, C. L., Robinson, F. B., Reynebeau, H. C., Kavouras, S. A., … Ganio, M. S. (2017). Effect of hypohydration on thermoregulatory responses in men with low and high body fat exercising in the heat. *Journal of Applied Physiology (Bethesda, Md.: 1985)*, *122*(1), 142–152. | **3** |
| Tustin, A. W., Cannon, D. L., Arbury, S. B., Thomas, R. J., & Hodgson, M. J. (2018). Risk Factors for Heat-Related Illness in U.S. Workers: An OSHA Case Series. *Journal of Occupational and Environmental Medicine*, *60*(8), e383–e389. | **3** |
| Tustin, A. W., Lamson, G. E., Jacklitsch, B. L., Thomas, R. J., Arbury, S. B., Cannon, D. L., … Hodgson, M. J. (2018). Evaluation of Occupational Exposure Limits for Heat Stress in Outdoor Workers—United States, 2011-2016. *MMWR. Morbidity and Mortality Weekly Report*, *67*(26), 733–737. | **3** |
| Vargas, N. T., Chapman, C. L., Johnson, B. D., Gathercole, R., Cramer, M. N., & Schlader, Z. J. (2019). Thermal behavior alleviates thermal discomfort during steady-state exercise without affecting whole body heat loss. *Journal of Applied Physiology*, *127*(4), 984–994. | **1** |
| Venugopal, V., Rekha, S., Manikandan, K., Latha, P. K., Vennila, V., Ganesan, N., … Chinnadurai, S. J. (2016). Heat stress and inadequate sanitary facilities at workplaces—An occupational health concern for women? *Global Health Action*, *9*, 31945. | **3** |
| Wang, F., Ferraro, S. del, Lin, L.-Y., Mayor, T. S., Molinaro, V., Ribeiro, M., … Holmér, I. (2012). Localised boundary air layer and clothing evaporative resistances for individual body segments. *Ergonomics*, *55*(7), 799–812. | **1** |
| Watkins, E. R., Hayes, M., Watt, P., & Richardson, A. J. (2018). Practical pre-cooling methods for occupational heat exposure. *Applied Ergonomics*, *70*, 26–33. | **3** |
| Watson, C., Troynikov, O., & Lingard, H. (2019). Design considerations for low-level risk personal protective clothing: A review. *Industrial Health*, *57*(3), 306–325. | **2** |
| Weiner, J. S. (1950). Observations on the working ability of Bantu mineworkers with reference to acclimatization to hot humid conditions. *British Journal of Industrial Medicine*, *7*(1), 17–26. | **3** |
| Westwood, C. S., Fallowfield, J. L., Delves, S. K., Nunns, M., Ogden, H. B., & Layden, J. D. (2021). Individual risk factors associated with exertional heat illness: A systematic review. *Experimental Physiology*, *106*(1), 191–199. | **1** |
| WHO \| Progress on household drinking water, sanitation and hygiene 2000-2017: (2019). Retrieved March 18, 2021, from https://www.who.int/water_sanitation_health/publications/jmp-report-2019/en/ | **3** |
| Yang, H., Cao, B., Ju, Y., & Zhu, Y. (2019). The effects of local cooling at different torso parts in improving body thermal comfort in hot indoor environments. *Energy and Buildings*, *198*, 528–541. | **2** |
| Yang, X., Wu, H., & Li, H. (2020). Dehydration-associated chronic kidney disease: A novel case of kidney failure in China. *BMC Nephrology*, *21*(1), 159. | **3** |
| Yeargin, S. W., Casa, D. J., Armstrong, L. E., Watson, G., Judelson, D. A., Psathas, E., & Sparrow, S. L. (2006). Heat acclimatization and hydration status of American football players during initial summer workouts. *Journal of Strength and Conditioning Research*, *20*(3), 463–470. | **3** |
| Zhao, M., Gao, C., Li, J., & Wang, F. (2015). Effects of two cooling garments on post-exercise thermal comfort of female subjects in the heat. *Fibers and Polymers*, *16*(6), 1403-1409. | **3** |
| Ziglio, E., & Alder, M. (1996). Gazing into the oracle: The Delphi method and its application to social policy and public health. | **3** |

*LOE, level of evidence. 14  Ebell MH, Siwek J, Weiss BD, *et al.* Strength of Recommendation  Taxonomy (SORT): A

 Patient-Centered Approach to Grading Evidence in the Medical Literature. *AFP* 2004;**69**:548.
